# Discovery of Semi- and Fully-Synthetic Carbohydrate Vaccines Against Bacterial Infections Using a Medicinal Chemistry Approach: Focus Review

**DOI:** 10.1021/acs.chemrev.0c01210

**Published:** 2021-04-01

**Authors:** Peter H. Seeberger

**Affiliations:** Department of Biomolecular Systems, Max Planck Institute of Colloids and Interfaces Am Mühlenberg 1, 14476 Potsdam, Germany; Institute of Chemistry and Biochemistry, Free University of Berlin, Arnimallee 22, 14195 Berlin, Germany

## Abstract

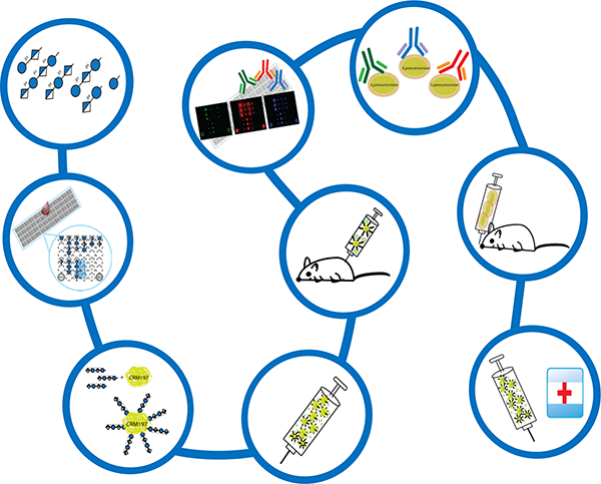

The glycocalyx, a thick layer of carbohydrates, surrounds the cell wall of most bacterial and parasitic pathogens. Recognition of these unique glycans by the human immune system results in destruction of the invaders. To elicit a protective immune response, polysaccharides either isolated from the bacterial cell surface or conjugated with a carrier protein, for T-cell help, are administered. Conjugate vaccines based on isolated carbohydrates currently protect millions of people against *Streptococcus pneumoniae*, *Haemophilus influenzae* type b, and *Neisseria meningitides* infections. Active pharmaceutical ingredients (APIs) are increasingly discovered by medicinal chemistry and synthetic in origin, rather than isolated from natural sources. Converting vaccines from biologicals to pharmaceuticals requires a fundamental understanding of how the human immune system recognizes carbohydrates and could now be realized. To illustrate the chemistry-based approach to vaccine discovery, I summarize efforts focusing on synthetic glycan-based medicinal chemistry to understand the mammalian antiglycan immune response and define glycan epitopes for novel synthetic glycoconjugate vaccines against *Streptococcus pneumoniae*, *Clostridium difficile*, *Klebsiella pneumoniae*, and other bacteria. The chemical tools described here help us gain fundamental insights into how the human system recognizes carbohydrates and drive the discovery of carbohydrate vaccines.

## 1 Introduction

Humans have been living with and fighting infectious diseases from the dawn of mankind. Still today, infectious diseases caused by bacteria, viruses, and parasites remain a major global health problem as vividly illustrated by the COVID-19 pandemic. The 20th century saw breakthroughs in ways to curb and control infectious diseases by fundamentally advancing two strategies. In the first strategy, antibiotics, antiviral, and antiparasitic medications were discovered to treat patients, and these compounds helped to save many millions of lives each year. In the second strategy, many vaccines were developed to protect people against some of the most serious infectious diseases. Vaccines prevent the formation of sequelae such as mental retardation that used to result in immense health care expenditures. Therefore, vaccination is the most cost-effective means for society to save lives. Thanks to the advent of vaccines, smallpocks and polio were almost eradicated in the 20th century. Many deadly infections, such as those caused by tetanus that repeatedly resulted in death after small injuries, are almost entirely forgotten today.

At the beginning of the 21st century many “new” infectious diseases such as corona viruses and drug resistant bacteria such as *Klebsiella pneumoniae* are threatening us while diseases we thought to have under control are re-emerging such as *Mycobacterium tuberculosis*. After almost a century of using effective antibiotics against most deadly bacterial infections, antibiotic resistance and the evolution of multiresistant bacteria that spread, particularly in hospital and long-term care settings, have become a major problem. The need to develop effective vaccines against many different infectious diseases caused by viruses, parasites, and bacteria is more urgent today than at any time since the development of the polio vaccine.^1^ With a greatly improved understanding of the human immune system and pathogen biology, the stage is set to create new vaccines to offer protection from infectious diseases that have become resistant to antibiotics. Synthetic chemistry is the enabling technology that supports the creation of efficacious and affordable vaccines.

### 1.1 Pharmaceuticals and Biologicals

The development of the pain medication Aspirin is prototypical for the process of drug discovery. Sumerian and Egyptian texts dating back 5000 years describe the leaves of the willow tree as a treatment for fever, inflammation, and aches. The active pharmaceutical ingredient, acetylsalicylic acid, was first isolated in 1828 by extracting the active ingredient from willow trees, producing bitter tasting yellow crystals that were named salicin. In 1853, treatment of sodium salicylate with acetyl chloride produced acetylsalicylic acid.^2^ The chemical structure was determined and more efficient syntheses were developed. The first rigorous clinical trial of salicin in 1876 confirmed its effects in reducing fever and joint inflammation in rheumatism patients. In 1899, the Bayer Company registered the trade name Aspirin for acetylsalicylic acid as a replacement for salicylate medicines.^3^ Thus, a plant extract, used as medication for thousands of years, was replaced by a synthetic pharmaceutical after the active pharmaceutical ingredient (API) was discovered and a synthesis was developed.

Most medications used today are synthetic molecules of defined composition. Regulatory agencies can implement strict measures to ensure proper characterization by spectroscopic and other means. The intellectual property is protected by composition of matter claims to defend novel medications from competition. Artemisinin combination therapies, the most effective means of treating malaria, stand out among the few major medications still derived by extraction. The natural product artemisinin, extracted from sweet wormwood plants, is converted to its artemether and artesunate derivatives, the major APIs.^4^

Regulatory agencies go to great lengths to ensure that marketed medications are pure, unadulterated, and exactly what is claimed. These agencies also oversee the production of vaccines that have not yet reached the same level of molecular definition as pharmaceuticals. In 1796, the British doctor Edward Jenner protected children from smallpox infections by injecting them with cowpox he obtained by draining pus from the cow blisters. Similar techniques had been practiced in China and other countries already for centuries. Although using children to test a medical hypothesis is utterly unthinkable today, his experiment marked the birth of vaccines. Whole cell vaccines were the first vaccines developed and consist of either attenuated or killed organisms. Although whole cell vaccines confer long-lasting immunity against infectious disease, culturing pathogenic organisms in high quantities can be difficult.^5^ In rare cases, attenuated pathogens might even cause disease in immunocompromised individuals.^6^ Subunit vaccines, often considered safer than whole cell vaccines, are preparations of molecularly defined components of the target organisms that cannot cause disease. The components can be proteins such as secreted toxins that can be obtained by recombinant expression,^5^ as well as virus-like particles or cell surface glycans. Due to the low immunogenicity of these antigens, subunit vaccines are usually formulated with adjuvants.^6^

Proteins are the antigens of choice in subunit vaccines due to their ability to induce an efficient immune response. However, protein antigens may fail to protect from disease when they are not exposed on the surface of a pathogen and therefore inaccessible to the immune system. The variability of surface proteins may prevent the buildup of a protective immunological memory and surface proteins may be very similar to or derived from cellular proteins of the host and hence may not be sufficiently immunogenic. Carbohydrate-based vaccines present an attractive alternative in these cases. Successful glycoconjugate vaccines made from isolated capsular polysaccharides (CPS) from bacteria as well as recombinantly expressed proteins identified from gene sequences (“reverse vaccinology”) have been developed.^5^

The logical end point in vaccine development, in analogy to the evolution in pharmaceuticals, is fully synthetic vaccines through which one or more synthetic antigens are administered. Hence, synthetic molecules described by their composition of matter would be used instead of biologicals defined by the production process. Then, regulatory authorities could define vaccine composition and purity based on physical characterization such as NMR spectroscopy and mass spectrometry; that is, these standard criteria for the regulatory approval practice of pharmaceuticals could be readily adopted to the vaccine field.

### 1.2 Carbohydrate Conjugate Vaccine Anatomy

Infection with pathogenic bacteria results in a potent immune response that is generated by the molecular recognition of proteins, lipids and carbohydrates. Proteins are thymus-dependent antigens that can induce the formation of immunological memory, while polysaccharide- or lipid-based thymus-independent antigens cannot elicit immunological memory. The goal of carbohydrate-based vaccines is the creation of a strong, specific and long-lasting immune response that is able to destroy the pathogen quickly with the help of B- and T-cells. Polysaccharide vaccines, however, consist exclusively of T-independent antigens, and thus cannot efficiently stimulate neonatal B cells.

In 1923, Heidelberger discovered that the immunodominant type-specific substance of *Streptococcus pneumoniae* is a polysaccharide^7^ before it was realized that the conjugation of monosaccharides to proteins enables the generation of saccharide-specific antibodies in vivo.^8,9^ CPS from *S. pneumoniae* were found to induce specific protection against the pathogen and this represented the advent of polysaccharide vaccines.^10^ Chemical attachment of type III pneumococci CPS to proteins from horse serum in 1931 allowed for successful vaccination of rabbits with these conjugates. Thus, the proof-of-principle for glycoconjugate vaccine efficacy against pathogenic bacteria was established 90 years ago.^11^

#### 1.2.1 Polysaccharide Vaccines

In the 1970s, polysaccharide vaccines were licensed against *Neisseria meningitidis* and *S. pneumoniae*. The first pneumococcal polysaccharide vaccine contained CPS from 14 different serotypes followed by a 23-valent formulation, along with further polysaccharide vaccines against pathogenic bacteria.^12^ Despite the remarkable success of polysaccharide vaccines in preventing invasive disease, polysaccharides were not effective in protecting infants with their immature immune systems from pathogens.^13^ While there is no clear-cut age at which the immune system starts to produce antibodies against T-independent antigens, it is believed that polysaccharide vaccines are not effective in infants under the age of 24 months.^14^ The *Haemophilus influenzae* type b (Hib) polysaccharide vaccine introduced in 1985 was withdrawn from the market in 1988 and replaced by CPS-protein conjugate vaccine formulations (see section 1.2.2).^15^ The pneumococcal polysaccharide vaccine PPV-23 (Pneumovax23, Merck) is recommended for children older than two years.^16^ Polysaccharide vaccines against *N. meningitidis* (Menomune, Sanofi Pasteur) and *Salmonella enterica* sv. Typhi (Typhim Vi, Sanofi Pasteur) are currently marketed but not recommended for infants.

#### 1.2.2 Carbohydrate Conjugate Vaccines

To overcome the immunogenicity problems with polysaccharides in young infants and the elderly, the two main groups at risk, and to improve the immunogenicity of carbohydrate epitopes by inducing a T-cell mediated immune response, the classical approach of conjugating polysaccharides to proteins was used. Tetanus toxoid (TT), diphtheria toxoid (DT), or cross-reacting material (CRM 197) a detoxified variant of DT have been used as carrier proteins.^17^ Upon processing by dendritic cells, the glycan epitopes are presented to T-helper cells by the major histocompatibility complex (MHC). Both cellular and cytokine-mediated signals then induce maturation and proliferation of the glycan-specific B cells and a memory response. High-affinity antibodies toward CPS and immunological memory are induced even in children under the age of two.^18,19^ Glycoconjugate vaccines have helped to significantly decrease the incidence of invasive diseases caused by pathogenic bacteria. Today, glycoconjugate vaccines constitute a multibillion dollar market and are included in immunization programs recommended by health authorities worldwide.^20^

Carbohydrate conjugate vaccines have almost completely replaced polysaccharide vaccines. Conjugate vaccines against Hib are a part of pentavalent vaccine combinations.^15^ Pneumococcal conjugate vaccines have been developed to cover an increasing number of serotypes, and current formulations are 10- (Synflorix, GSK) and 13-valent (Prevnar13, Pfizer).^21^ Conjugate vaccines against pneumonia have been an immense medical and commercial success. Novel glycoconjugate vaccines against a variety of pathogens are in development.^22,23^

Most marketed glycoconjugate vaccines contain saccharides isolated from cultured bacteria. Bacteria are killed by heat or chemical treatment prior to CPS precipitation. Purification of CPS by ultracentrifugation, gel permeation, enzyme treatment and ultrafiltration results in polysaccharides with less than 3% nucleic acid and protein contaminations.^24^ Purified CPS are depolymerized by microfluidization or enzymatic degradation and the size distribution of the fragments is determined by size exclusion chromatography and many other techniques.^25^

CPS fragments are chemically activated for conjugation to the carrier protein.^26^ Single point attachment utilizes one reactive functional group per CPS fragment, typically the anomeric carbon of the reducing end monosaccharide, to connect it via reductive amination to a lysine side chain of the carrier.^27^ Single point attachment methods yield “neoglycoconjugates” that stand in contrast to methods that introduce multiple reactive groups per CPS fragment, leading to cross-linked carrier-glycan lattices. For example, multipoint attachment can be performed via carbodiimide coupling of uronic acids and cyanogen bromide activation of vicinal hydroxyl groups.^28−30^ Alternatively, periodate oxidation of saccharides generates aldehyde groups that engage in reductive amination.^31^ Conjugation via thioether formation between thiolated polysaccharide fragments and haloacylated carrier proteins ensures chemoselective couplings.^32,33^ The choice of the conjugation method has to be matched to the functional groups available on the carrier protein. Toxins like DT and TT are usually inactivated by treatment with amine-reactive reagents like formaldehyde. Inactivation reduces the availability of reactive lysine residues, and therefore toxoid carriers are preactivated and spacer molecules are incorporated during conjugation.^34^ It must be assessed whether any reactive groups remain in the conjugate. The saccharide-to-protein ratio must be reproducible for different vaccine lots and the amount of free, unconjugated glycan chains must be determined.^35^

Adjuvants are immunostimulatory components that are added to the vaccine to improve the immune response. For human use, mainly aluminum salts such as aluminum phosphate or aluminum hydroxide are added.^36^ The vaccine formulation contains the glycoconjugate that is adsorbed onto the aluminum salt. The formulated product is either stored as a lyophilized powder or as a suspension. The vaccine has to be kept cold at all times in order to avoid denaturation of the protein and potentially harmful side effects. The cost of cold chain maintenance can represent as much as half of the overall cost. All current glycoconjugate vaccines have to be delivered by injection, which complicates administration in developing countries.

#### 1.2.3 Semisynthetic Carbohydrate Conjugate Vaccines

Despite the immense medical and commercial success of conjugate vaccines based on isolated polysaccharides, their development was challenging and took decades to accomplish. The procurement of glycans for the production of conjugate vaccines reaches its limits in cases where the pathogen cannot be cultured or the isolation of the glycan is too difficult. Not every bacterial strain can be cultured easily. Even if a strain can be cultured, the production of the correct CPS in sufficient quantities requires careful process optimization.^37,38^ Some labile polysaccharides may decompose during isolation.^39,40^ Very stable polysaccharides such as the CPS on *Salmonella typhii* may be difficult to depolymerize.^41^ Isolated polysaccharide preparations inevitably contain small amounts of impurities, such as the pneumococcal C-polysaccharide that are not found in synthetic glycans.

Methods to synthesize the glycan epitope have been explored since the late 1980s.^42−47^ A semisynthetic glycoconjugate vaccine (QuimiHib) was developed in Cuba and is marketed in several South American countries.^48^ In this case, not a single glycotope was used but rather a mixture of oligosaccharides of different length that were prepared by polymerization of a synthetic building block. Conjugation of the synthetic glycans to TT-carrier protein yielded a semisynthetic glycoconjugate vaccine that has proven highly effective since its introduction over a decade ago. To date, no other semisynthetic vaccine has been marketed, owing to a bias in the vaccine industry against the adoption of new technologies.

#### 1.2.4 Fully Synthetic Carbohydrate Conjugate Vaccines

**Figure 1:**
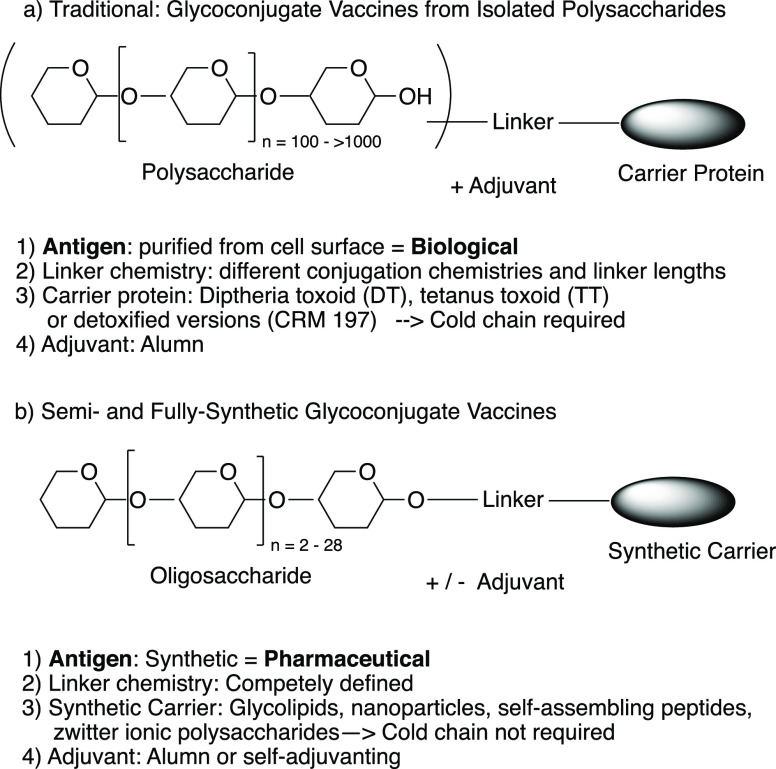
Glycoconjugate vaccine concept; (a) The traditional approach and (b) the semisynthetic or fully synthetic glycoconjugate vaccine approach.

Conceptually, molecularly completely defined, fully synthetic carbohydrate-conjugate vaccines are desirable. Synthetic oligosaccharide antigens will replace isolated polysaccharides, a synthetic carrier such as a glycolipid or synthetic peptide may substitute an expressed protein and ideally synthetic constructs would no longer require an adjuvant (Figure 1). Such fully synthetic vaccines would be pharmaceuticals rather than biologicals. While the creation of such vaccines is conceivable, none of these vaccine candidates has yet gone past the exploratory stage into clinical trials.^49^

### 1.3 Synthetic Carbohydrate Vaccine Development Process

**Figure 2:**
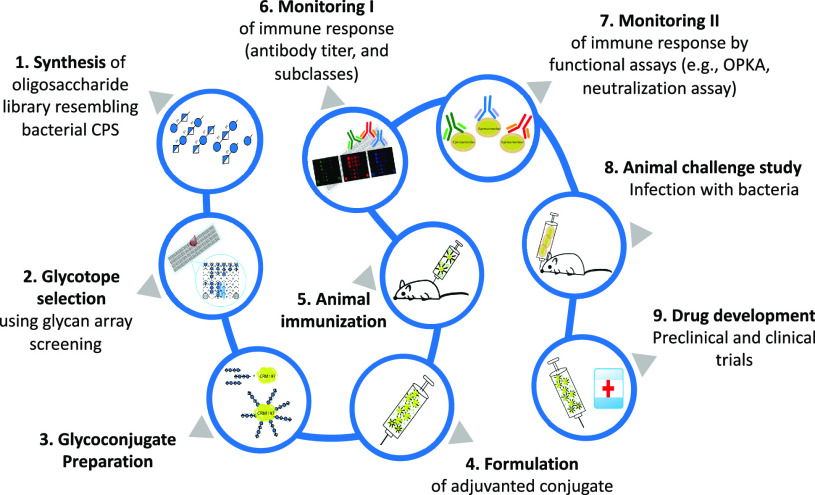
Overall process of synthetic carbohydrate vaccine development.

A medicinal chemistry approach toward glycoconjugate vaccine development requires a fundamental understanding of how the human immune system recognizes cell-surface glycans and how this recognition process results in a B-cell and T-cell response that in turn induces the killing of bacterial pathogens. A multidisciplinary effort of chemists, immunologists, and eventually structural biologists needs to focus on the identification of a specific, protective oligosaccharide as part of the synthetic carbohydrate vaccine development process (Figure 2).

#### 1.3.1 Selecting the Target Disease

Any pathogen that carries unique glycans on its surface is in principle a target for the development of glycoconjugate vaccines or treatment with monoclonal antibodies. Most Gram-negative bacteria as well as many protozoan parasites carry signature glycoconjugates on their cell surface. In selecting a vaccine target, the medical need and insights concerning the cell-surface glycans are important. Ideally, the structures of the pathogen cell surface glycans in question are known and the chosen glycan is unique and, to avoid autoimmunity, differs from any human cell-surface glycans.

##### 1.3.1.1 *Streptococcus pneumoniae*

*S. pneumoniae* one of the most important human pathogens, is endemic globally and the most common cause of acute otitis media, sinusitis, pneumonia, and bacterial meningitis. In developing countries, pneumonia is a serious pediatric disease, and it is estimated that more than a million children below the age of five die each year from pneumococcal pneumonia.^50^ Initially, unconjugated polysaccharides were used for vaccination (Pneumovax 23 (PPV-23) from Merck), but these preparations suffered from poor immunogenicity. *S. pneumoniae* carbohydrate conjugate vaccines have become blockbusters. The 13-valent glycoconjugate vaccine Prevnar-13 from Pfizer dominates the market over ten-valent Synflorix from GSK. These glycoconjugate vaccines have dramatically reduced the number of deaths due to *S. pneumoniae* infections. The existence of more than 90 different serotypes renders development of a universal vaccine challenging.^51^

Currently available pneumococcal vaccines were based on epidemiological and prevalence data for North America and Europe. Many of the serotypes that are prevalent in developing and emerging nations are missing in these vaccines. Therefore, some national health agencies are reluctant to introduce the vaccine into their national vaccination programs. Moreover, the immune response induced against each serotype varies greatly. This can be attributed to differences in chain length, epitope number, and distribution. Since these vaccines are based on isolated polysaccharides, reproducibly controlling glycan chain length is very difficult.

##### 1.3.1.2 *Haemophilus influenzae* type b (Hib)

Hib infections are widespread throughout the world and may develop under various scenarios with meningitis being the most frequent outcome. Worldwide, Hib infections account for three million cases of severe illness and 400 000 deaths annually, with a peak of incidence among infants of age 4 to 18 months.^52^ Following colonization of the pharynx, the Gram-negative bacterium may enter the bloodstream and subsequently spread to reach various target organs resulting in different clinical forms of Hib disease including meningitis, pneumonia, and arthritis. Hib meningitis is often fatal (5–40% of cases depending on the country) and may lead to neurological sequelae such as deafness, motor deficit, or mental retardation. Medical management relies on intensive care and appropriate antibiotic therapy.

The Hib vaccine is usually administered along with the other vaccines included in the childhood vaccination schedule and has resulted in a rapid decline in the number of cases in industrialized countries but is not used everywhere.^53−55^ A semisynthetic glycoconjugate vaccine (QuimiHib) was developed in Cuba and is marketed in several South American countries.^48^ Some of the currently marketed Hib vaccines face stability issues as the Hib polysaccharide is not stable for long periods of time when formulated with alum. Therefore, the lyophilized vaccine has to be solubilized by the physician just prior to injection. Vaccine formulations that circumvent that stability issue have been commercially more successful in recent years.

##### 1.3.1.3 *Neisseria meningitidis*

*N. meningitidis* is a leading cause of bacterial meningitis and the only Gram-negative encapsulated bacterium responsible for large epidemics.^56^ Bacterial meningitis accounts for 1.2 million cases of the disease worldwide annually, affecting mostly infants, children, and young adults who do not have specific protective antibodies.^57^ Out of the 13 serotypes, the most prevalent are A, B, C, W135, and Y. Two types of polysaccharides, CPS and lipopolysaccharide (LPS), are the major virulence factors in *N. meningitidis* infections. There are currently several quadrivalent vaccines available to prevent meningococcal disease, all by targeting serogroups A, C, W-135, and Y. In addition to conjugate vaccines, a polysaccharide vaccine is being marketed.^58−60^ A protein-based vaccine for serotype B gained approval in 2013.^61^

##### 1.3.1.4 *Clostridium difficile*

The Gram-positive, spore-forming bacterium *C. difficile* is the most common cause of nosocomial diarrhea worldwide.^62^ The disruption of intestinal flora by antibiotic administration allows for colonization by and/or overgrowth of drug-resistant, toxin-producing *C. difficile* spores. The disease is mostly associated with hospital and long-term care facilities. Risk factors for *C. difficile* infections (CDIs) include broad-spectrum antibiotic use, hospitalization, and advanced age. Over 450 000 CDI cases cause about 30 000 deaths and over $4.8 billion medical costs annually in the U.S. alone. In recent years, infection and death rates have been increasing drastically. In addition to the main risk group, the elderly, children, young adults and pregnant women are now infected, thereby increasing the social and economic burden.^63^ The emergence of new *C. difficile* strains, such as ribotype 027 that has quickly spread with increased virulence, toxin production, and antibiotic resistance is partially responsible for this development.^64^ Vaccination as an alternative to antibiotic treatment is highly desirable. Toxin-neutralizing immunization can protect against lethal challenge with *C. difficile* in hamsters, but toxin-based vaccines cannot inhibit bacterial colonization, which precedes toxin production.^65^ Recurrent CDIs are serious clinical problems affecting about 20% of patients after cessation of therapy, either due to recolonialization by the same or reinfection with a different *C. difficile* strain.^66^ Preventing colonialization by vaccination against surface antigens may limit recurrence more effectively than toxin-neutralizing approaches. Several cell-surface glycans of *C. difficile* have been characterized.^67,68^

##### 1.3.1.5 *Klebsiella pneumoniae*

*K. pneumoniae* (Kp) is the leading cause of nosocomial respiratory and urinary tract infections, as well as bacteremia, primarily among newborns and immunocompromised patients.^69^ Carbapenem-resistant Kp (CR-Kp) are now commonly encountered in hospitals worldwide. Outbreaks occur with increasing frequency;^70−72^ strains spread quickly^73^ and cause high morbidity and mortality. Most clinical CR-Kp isolates contain a dominant strain, sequence type 258 (ST258), that expresses Kp carbapenemase. Efficacious vaccination of risk groups is direly needed as treatment options for CR-Kp are diminishing. Currently, there are no vaccines against Kp available.

##### 1.3.1.6 Group A Streptococcus

Group A Streptococcus (GAS) infections are a global concern as they cause a broad spectrum of diseases ranging from asymptomatic colonization and uncomplicated skin infections to life-threatening invasive illnesses including sepsis and toxic shock syndrome.^74^ Pharyngitis may lead to delayed sequelae such as rheumatic fever. While GAS has been on the WHO priority prevention list for decades, currently, no vaccine to prevent GAS infections exists.

##### 1.3.1.7 Shigella

Gram-negative, facultative anaerobic Shigella bacteria cause bacillary dysentery (shigellosis) for which there is no broadly available vaccine. It is estimated that every year 800 000 people, mainly children under five years of age in developing countries, die from diarrheal diseases.^75^*Shigellae* and enterotoxigenic *Escherichia coli* (ETEC) are also major causes of morbidity and mortality among older children, adolescents, and adults. Pathogenic *Shigellae* are highly infectious and are transmitted through person-to-person contact and contaminated food. An effective, multivalent vaccine against shigellosis that covers multiple species and serotypes is a global priority. Four major *Shigella* species are distinguished on the basis of the O-specific polysaccharide (O-SP) structure of their cell surface lipopolysaccharide. *S. flexneri*, with at least 15 serotypes and subserotypes, and *S. sonnei*, with only one serotype, are the species responsible for the vast majority of cases worldwide.^76^ Most Shigella vaccine candidates intend to induce an antibody response against the LPS that is both a virulence factor and a major surface protective antigen.^77^

##### 1.3.1.8 *Salmonella typhii*

Typhoid fever, caused by the Gram-negative bacterium *Salmonella enterica* serovar typhi, is closely associated with poor food hygiene and inadequate sanitation. After ingestion of contaminated food and water, Salmonellae penetrate the gut epithelium to spread to visceral tissues, including liver and spleen. Patients experience fatigue, headache, abdominal pain, fever, and constipation or diarrhea. Severe forms may entail cerebral dysfunction, delirium, and shock. *S. typhi* causes typhoid in humans.^78^

About 16 million cases of typhoid fever result in approximately 600 000 deaths globally every year. The incidence of typhoid fever peaks between the ages of 5 and 12 years in endemic areas. In recent years *S. typhi* has gradually acquired resistance to oral antibiotics. Nonavailability of drugs and developing drug resistance render an efficacious and affordable vaccine highly desirable.^79^

#### 1.3.2 Selecting the Cell-Surface Glycan Target

**Figure 3:**
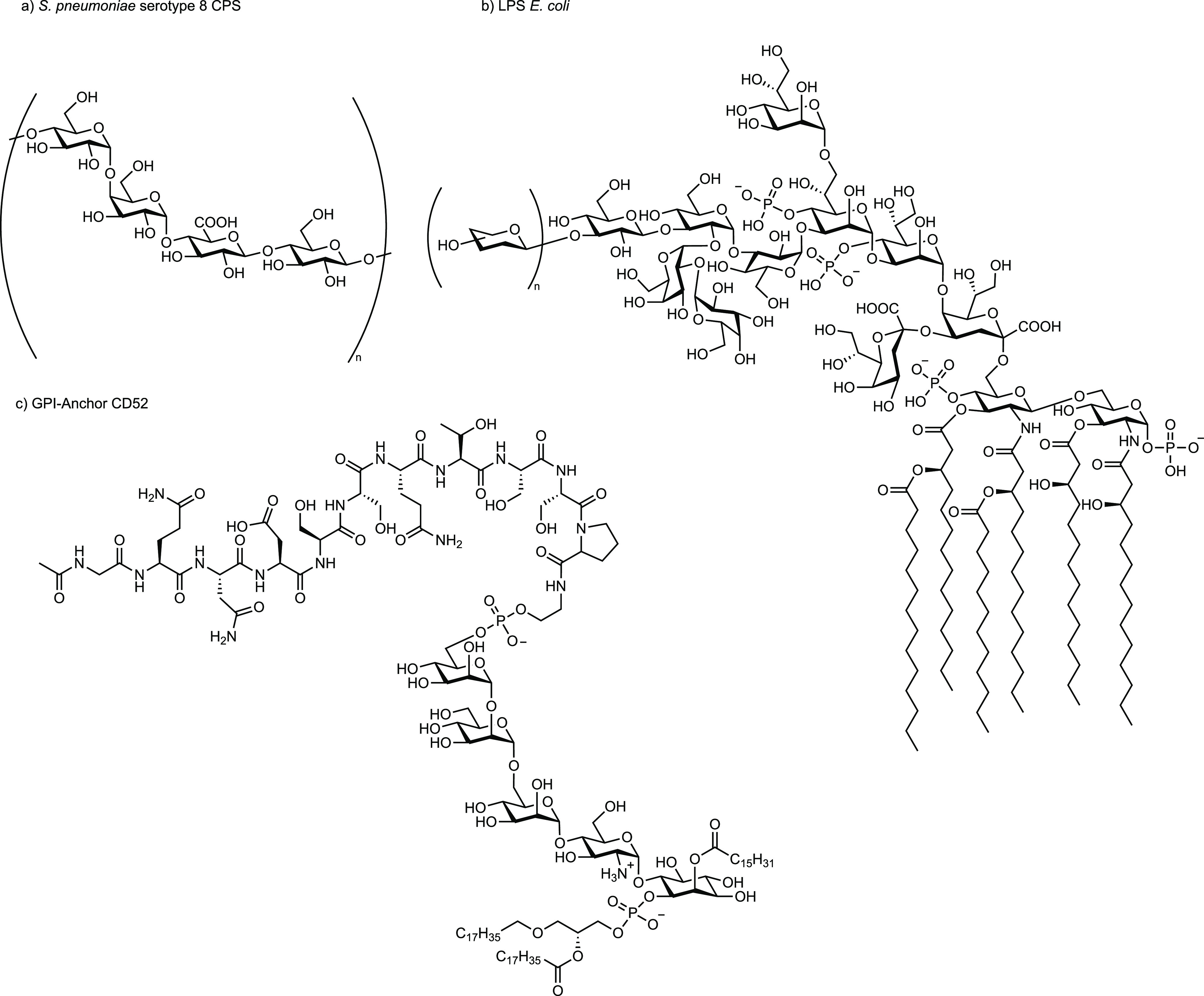
Schematic view of (a) capsular polysaccharide (CPS), (b) lipopolysaccharide (LPS), and (c) glycerophosphatidylinositol (GPI) anchors. n = number of repeating units.

Once a pathogen has been identified as a potential vaccine target based on a specific medical need, information concerning the composition of the cell-surface glycans present on the pathogen are essential. Many pathogenic bacteria, fungi, and protozoan parasites cover themselves with a layer of CPS, LPS, and related structures (Figure 3).^80^ Microorganisms use these capsules to prevent desiccation and escape from components of the innate immune system by shielding other cell surface antigens.^81^ Capsules are major virulence factors since they prevent complement activation and opsonization.^82^ Most CPS are biosynthesized in a modular manner from oligosaccharide precursors, and hence usually consist of repeating units (RUs).^83^

Knowledge concerning the structure of CPS, O-antigens, or glycolipids is gained by isolation of the cell-surface glycans from cultured bacteria followed by chemical degradation and detailed structural elucidation based on physical methods such as NMR spectroscopy. This provides insights concerning the size and the composition of the RU. The structural diversity of CPS of pathogenic bacteria is enormous.^84^ In *S. pneumoniae*, more than 90 different CPS RU structures are distinguished and serotypes assigned.^85^ RU length ranges from mono- to octasaccharides^86^ and the backbone in some cases comprises phosphodiester linkages, as is the case for *H. influenzae* type b. Polysaccharides may be linear or branched and contain common or rare sugars. While human cell surface glycans are composed of just nine different monosaccharides, the number of building blocks that make up the bacterial glycome is in the hundreds.^84^ Monosaccharides may be present in pyranose or furanose forms or as open chain polyalcohols. Covalent modification with groups such as acetate, phosphate, pyruvate, or glycerate further increases structural variability. Rare monosaccharides, unusual linkages, and modifications likely contribute to the nonself recognition of bacterial CPS by the human immune system.

**Figure 4:**
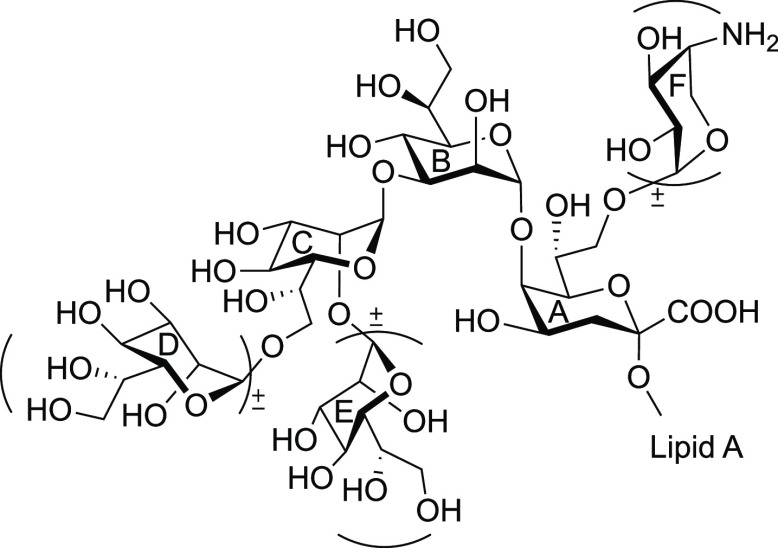
Conserved inner core oligosaccharides of LPS from *Y. pestis*, *H. influenzae*, and *Proteus*. (A) 3-Deoxy-α-d-manno-oct-2-ulosonic acid (Kdo); (B, C, D, and E) l-glycero-d-manno-heptose (Hep); (F) 4-amino-4-deoxy-β-l-arabinose (Ara4N).

The pathogenicity and virulence of Gram-negative bacteria are often associated with the LPS coat.^85^ LPS, a highly complex glycolipid, is the major component of the outer membrane of Gram-negative bacteria. LPS is comprised of the lipid A moiety, the core oligosaccharide, and the O-specific polysaccharide (Figure 3b). The core oligosaccharide, connecting lipid A with the outer O-antigen, is present in every natural LPS structure. The O-antigen in turn may be missing in many pathogenic bacteria such as *N. meningitidis* and *H. influenzae*. Structurally, the core region of LPS can be further subdivided into inner core and outer core. The main structural motifs of the inner core are highly conserved throughout the Gram-negative bacteria while the sugars of the outer core vary greatly between strains.^87^ 3-Deoxy-α-d-manno-oct-2-ulosonic acid (Kdo) and L -glycero-d-manno-heptose (Hep) are ubiquitously present in every LPS structure (Figure 4). Most Gram-negative bacteria share the trisaccharide l,d-Hep-(1 → 3)-l,d-Hep-(1 → 5)-Kdo as a common conserved inner core structure. This core can be decorated with other glycans, phosphates, or occasionally acetyl groups.

As a potent virulence factor, LPS also serves as a surface pathogen-associated antigen for recognition by the host immune system.^88^ Therefore, LPS has attracted much interest for the development of vaccine candidates.

Protozoan cell membranes are rich in glycerophosphatidylinositol (GPI) anchors that are often linked to cell surface proteins (Figure 3c). GPI anchors can activate components of the innate immune system, such as Toll-like receptors (TLR).^89^ Protozoan GPIs have been shown to induce the generation of specific antibodies and may thus be the antigens of choice for vaccination against these parasites.^90^ A fully synthetic GPI provided efficient protection against Plasmodium infections when conjugated to a carrier protein,^91^ while immunization with GPI-anchored proteins derived from the multicellular trematode *Schistosoma mansonii* protected mice from this parasite.^92^

#### 1.3.3 Synthetic Oligosaccharides as Basis for Glycotope Identification

When a disease target for vaccination has been identified and the composition of one or more unique glycans of the pathogen cell surface are known, a carbohydrate conjugate vaccine program commences with medicinal chemistry work focused on identifying the minimally protective epitope. The length of the antigen has to be determined, the frameshift of the RU has to be worked out, and the importance of unusual sugars that are not found in the human repertoire, as well as the importance of covalent modifications, have to be determined.

Ultimately, designing a carbohydrate from first-principles based simply on the knowledge concerning the composition of the cell surface glycan would be highly desirable. Currently, however, our understanding of what makes a glycan immunogenic and antigenic is too rudimentary to take a rational design approach. Therefore, series of synthetic glycans related to the cell-surface glycan target are prepared to serve as tools for the identification of a lead antigen. Since the isolation of specific glycans is difficult and cannot rely on amplification procedures, chemical and enzymatic syntheses, or combinations thereof, have served to procure these molecules.

##### 1.3.3.1 Traditional Synthesis of Oligosaccharide Antigens

Chemical solution-phase syntheses have helped to define minimal glycotopes for use in semisynthetic glycoconjugate vaccines since the 1980s.^28−30^ The fundamental challenges of glycan synthesis are protecting group manipulations and stereochemical control over glycosidic bond formation. The selective exposure of one hydroxyl group allows for regioselective addition of another (mono)saccharide unit.^93^ The choice of protecting groups and the order of protecting group installation are essential for a synthesis route to be successful. A major challenge in glycan synthesis is the stereoselective formation of glycosidic bonds. A variety of methods are available to stereoselectively generate glycosidic linkages. The yield and the stereochemical outcome of these reactions depend on the steric and electronic nature of the glycosylating agent (the glycosyl donor), as well as the nature of the nucleophile and the reaction conditions chosen.^93^

##### 1.3.3.2 Automated Glycan Assembly of Oligosaccharide Antigens

**Figure 5:**
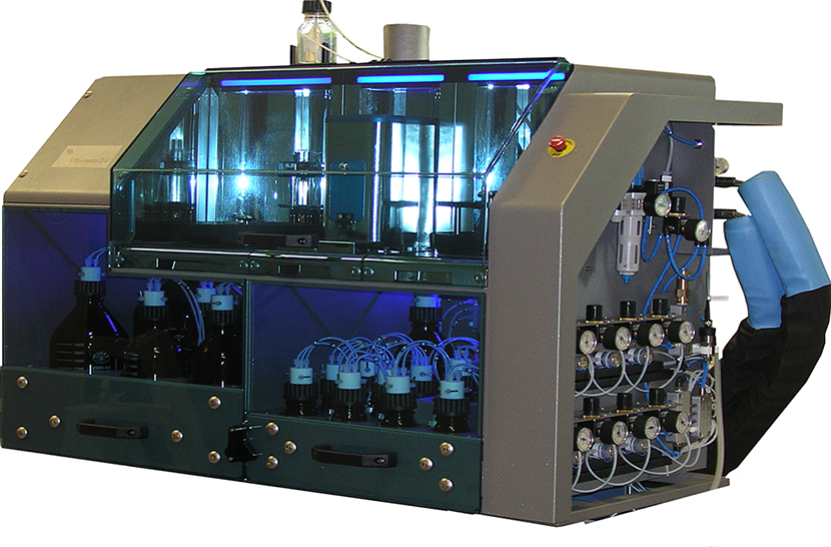
First commercially available automated oligosaccharide synthesizer.

The chemical synthesis of defined carbohydrates remained challenging and time-consuming at a time when automated peptide^94^ and oligonucleotide^95^ syntheses had become routine. The concept of automated glycan assembly (AGA) was introduced in 2001^96^ and systematically improved over the following two decades. Today, synthetic glycans as long as 100-mers^97^ have been assembled by AGA.^98^ The streamlined AGA process can rely now on a commercial Glyconeer 2.1 automated oligosaccharide synthesizer (Figure 5) and commercially available monosaccharide building blocks to rapidly assemble conjugation-ready oligosaccharides.^99^ Rapid access to defined carbohydrate antigens via AGA has drastically accelerated the medicinal chemistry approach by preparing collections of oligosaccharides quickly.

#### 1.3.4 Glycan Microarray Analysis of Sera to Identify Antigen Hits

The classical approach to assess the antigenicity of oligosaccharides involves immunization trials with the glycoconjugates of interest. The affinities of antisera from immunized animals to conjugated oligosaccharides and native CPS are determined.^100^ Antisera or monoclonal antibodies are tested for their ability to bind pathogens and promote phagocytosis.^101,102^ More recent approaches of epitope mapping employ screening of antibodies from infected or immunized individuals for binding to synthetic oligosaccharides.^103^ Uncovering a potential antigenic polysaccharide epitope by means other than immunization can help to reduce the number of required animal trials.

**Figure 6:**
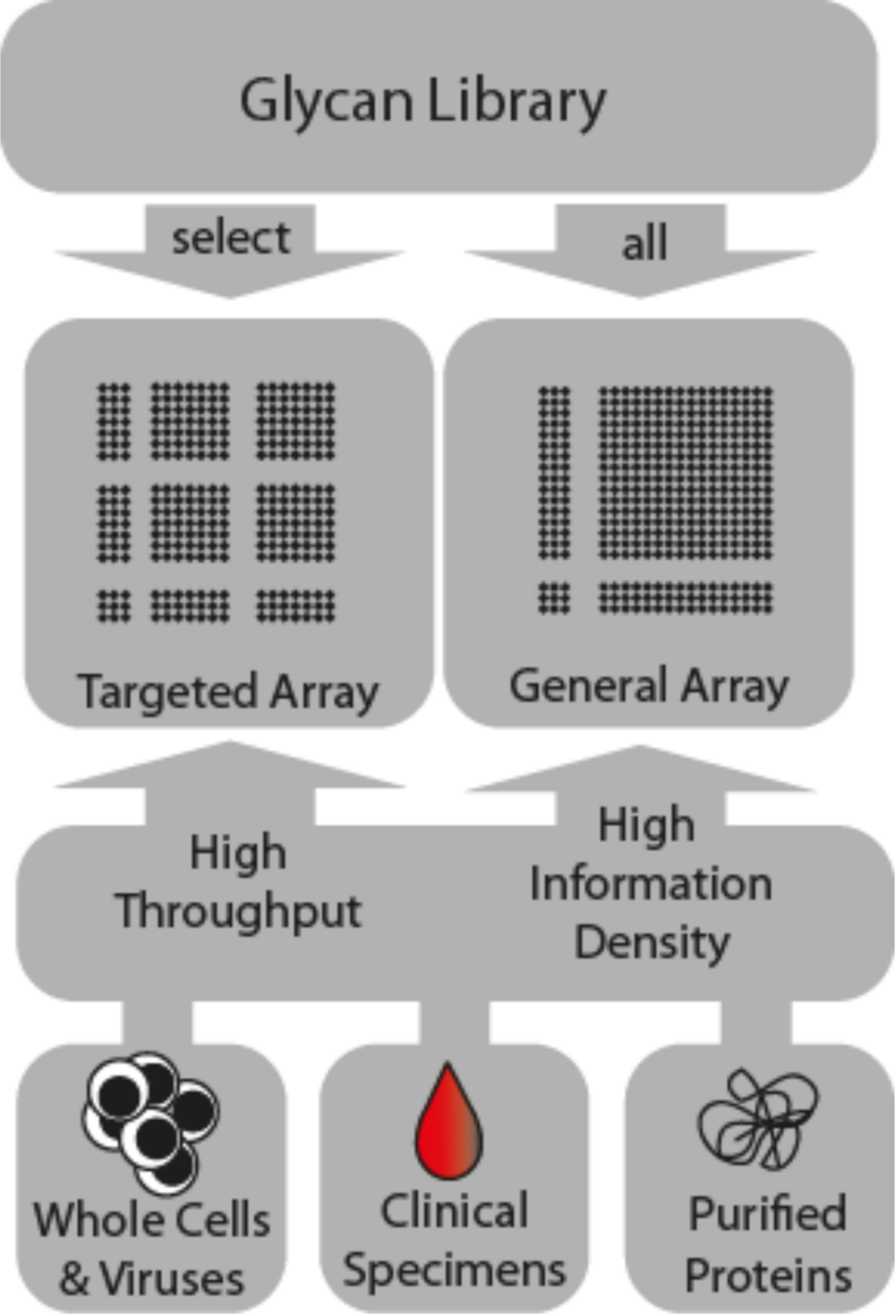
Glycan microarray screening of protein-carbohydrate interactions. Based on the experimental design, different glycans are printed onto standard microarray slides. Special targeted arrays are designed to contain glycan subsets for high throughput screening, such as serum analysis.

Carbohydrate microarrays that carry hundreds of different sugars bound covalently in small spots on surfaces are now a standard tool for glycobiologists (Figure 6).^103^ The miniaturized array methodology is well suited for serological investigations as only tiny amounts of both glycan and blood serum are required and many binding events can be screened in parallel. Access to defined bacterial glycans has been the bottleneck for the use of glycan arrays. With accelerated methods to assemble bacterial oligosaccharides, screening of patient samples such as blood and stool with the help of glycan microarrays has been greatly facilitated.

Screening clinical blood or stool samples using glycan arrays provides insights into the antigenicity of cell-surface glycan epitopes by detecting the presence of antibodies (in samples from patients compared to healthy controls) that recognize synthetic glycans resembling cell-surface RUs and related oligosaccharides. When a bacterium colonizes the lungs and is found in the blood, as is the case for *S. pneumoniae*, IgM and IgG antibody levels are determined. For gut bacteria such as *C. difficile*, the interaction with IgA antibodies is assessed.^104^ If a correlation between the presence of antibodies and disease outcome can be established, such that patients with antibodies show milder forms of the disease, this indicates a good lead for the development of a carbohydrate based vaccine. Microarrays of synthetic GPI glycans helped to demonstrate that adults in endemic areas are protected from severe malaria by antiglycan antibodies.^105^ Similarly, the GPI anchor of *T. gondii* was identified as a diagnostic marker for toxoplasmosis.^106^

#### 1.3.5 Determination of the Minimal Glycotope and Selection of Oligosaccharides for Immunological Evaluation

Screening of sera from patients as well as reference sera provides insights into the potential glycotope. To obtain a molecular level picture, a series of oligosaccharides related to the glycans bound by the serum antibodies is prepared. In some cases, protective monoclonal antibodies have been identified against a disease and the synthetic oligosaccharides can serve to provide insights into the nature of a protective glycotope. The oligosaccharides differ in length, frameshift, terminal monosaccharide, and covalent modifications (thus can also differ in net charge). When potentially labile functional groups are found within the glycotope, stable analogues may be tested as well.

The synthetic oligosaccharides are equipped with a unique terminal functional group such as an amine or a thiol that facilitate covalent attachment to the surface of arrays and to carriers for immunological studies. Binding of monoclonal antibodies to different oligosaccharides is tested on glycan arrays. The interactions are tested for specificity using native CPS or LPS to block the interaction. This process identifies one or more candidates for further immunological evaluation.^103^

#### 1.3.6 Preparation of Glycoconjugates

Since most glycans cannot induce a T-cell-mediated immune response, the carbohydrate antigen has to be connected to a carrier. Currently marketed vaccines contain as carrier protein diphtheria toxoid (DT), tetanus toxoid (TT) or a detoxified version thereof, such as CRM197. These carrier proteins are isolated following either homologous or heterologous expression in bioreactors, using well-characterized bacterial strains.^107^ The proteins are purified by chromatography, sterile filtered and analyzed for homogeneity and structure.^108,109^ A host of methods for the preparation of carbohydrate–protein conjugates have been developed and proper conjugation chemistry is key to efficient glycoconjugate production.^110^ A linker must be selected that induces minimal undesired immunogenic responses. For example, thiol modified oligosaccharides can be readily conjugated to maleimide-containing CRM197 in phosphate buffer at room temperature to afford glycoconjugates. Alternatively, synthetic oligosaccharides containing a terminal amine are converted into the *p*-nitrophenyladipate ester^111^ derivative and covalently coupled to, for example, lysine side chains in CRM197.

Ideally, the number of oligosaccharides conjugated to each CRM197 protein is calculated from the mass shift measured using MALDI-TOF MS. Typically, between four and ten of the 40 lysine residues available in CRM197 are coupled to an oligosaccharide.^112^ Further characterization of the glycoconjugates is performed using SDS-polyacrylamide gel electrophoresis (SDS-PAGE) confirming an increase in molecular weight. Glycan content determination to confirm the loading from MALDI is based on high performance anion exchange chromatography with pulsed amperometric detection (HPAEC-PAD) after alkaline hydrolysis.^113−115^

Many new carrier strategies focus on multivalent hapten presentation. Encouraging results in vaccination experiments have been obtained with oligosaccharide-bound virosomes, liposomes and gold nanoparticles (GNP).^116^ The fate of fully synthetic carriers, such as GNP, inside an organism is often unclear. Thus, both *in vivo* toxicity and clearance pathways have to be accurately assessed for these materials.

#### 1.3.7 Glycoconjugate Formulation

Covalent attachment of carbohydrate antigens to carrier proteins produces neoglycoconjugates that, unlike native carbohydrates, induce a T cell-dependent immune response. The addition of immunostimulatory substances, adjuvants, helps to boost the immune system to provide a strong response. Glycoconjugates are often formulated either with Alum (Alhydrogel; aluminum hydroxide) or Freund’s Adjuvant (FA). Alum is an adjuvant that is approved for use in human vaccines. Freund’s adjuvant is an effective adjuvant in mice that has been successfully employed to raise antibodies to a synthetic oligosaccharide antigen but is not allowed to be used in humans due to its toxicity.^117^ Finally, after formulating the conjugate with an adjuvant, the stability of the glycoconjugate may be assessed.,^15^,^107^^118^

#### 1.3.8 In Vivo Immunogenicity Tests

To test the immunogenicity of glycoconjugates, either mice or rabbits are used. Mice are convenient as they are inexpensive and easy to keep. The immune system of rabbits is however closer to that of humans and the immunological results that are obtained are a better predictor for a human response. Groups of mice or rabbits are immunized subcutaneously with doses of glycoconjugate formulated either with FA or Alum. An immunization schedule follows a prime-boost regime whereby the formulated glycoconjugate is subcutaneously injected up to three times at 14 day intervals.

The antihapten antibody titers are monitored using glycan array analysis. The immunogenicity of synthetic antigens is often strongly dependent on the adjuvant formulation. Conjugates formulated in FA often induce higher antibody titers in mice when compared to Alum formulated conjugates. Microarrays also include CRM197 to assess antibody responses to the carrier protein and the generic spacer moiety. IgG, IgM, and IgA isotype antibodies to the oligosaccharide antigen are detected to assess whether immunoglobulin class switching is induced. Freund’s adjuvant often elicits higher antibody levels than Alum.^119^ Nonadjuvanted glycoconjugates may be immunogenic as well, but induce a weaker and shorter-lived antibody response. At a given time point, IgG levels are expressed as mean fluorescence intensity (MFI) and compared between different adjuvanted groups.

Glycan microarrays assist in epitope mapping and identify its minimum size. Antibodies raised using a particular glycoconjugate often not only recognize the antigen used for immunization but also related shorter oligosaccharides. The epitope recognition pattern in animals immunized with glycoconjugates formulated with different adjuvants often differ.^119^

To gain further insights into the nature of the humoral immune response to the epitopes, and the differences observed in mice immunized with different adjuvants, binding of serum antibodies to epitopes can be analyzed by surface plasmon resonance (SPR). Antibodies in the sera may show increasing binding and stability values over time, indicating affinity maturation to epitopes during the course of the immune response.

In many cases, especially for Hib vaccines, Zika rabbits are the animal model of choice. As for mice, groups of rabbits are immunized in a prime-boost regime with glycoconjugate formulated with adjuvant. The analysis of the immune response in rabbits follows the protocols developed for mice.^120^

#### 1.3.9 Monoclonal Antibodies Against Synthetic Epitopes

Understanding how complex carbohydrates interact with antibodies is a first step toward establishing rules for carbohydrate antigen design. The detailed analysis of a monoclonal antibody was performed for a tetrasaccharide component of the Bacillus collagen-like protein of anthracis (BclA) glycoprotein, found on the surface of the spores of *Bacillus anthracis*, the agent that causes the acute zoonotic disease, anthrax. A tetrasaccharide-conjugate elicited IgG antibodies that bind specifically to native *B. anthracis* endospores.^121^ These antibodies were the basis for a commercial diagnostic anthrax test. Crucial antibody-binding positions on the sugar antigen were identified using a combination of synthetic glycan microarray screening, surface plasmon resonance (SPR), and saturation transfer difference (STD) NMR analysis.^121^

To uncover the structural elements of the carbohydrate that influence this selectivity, microarray screening was performed using a family of synthetic oligosaccharides related to the original BclA tetrasaccharide. These synthetic glycans were screened for their ability to bind the antidisaccharide and antitetrasaccharide mAbs. Glycan array screening revealed anthrose is the minimal unit required for binding antidisaccharide mAbs.

**Figure 7:**
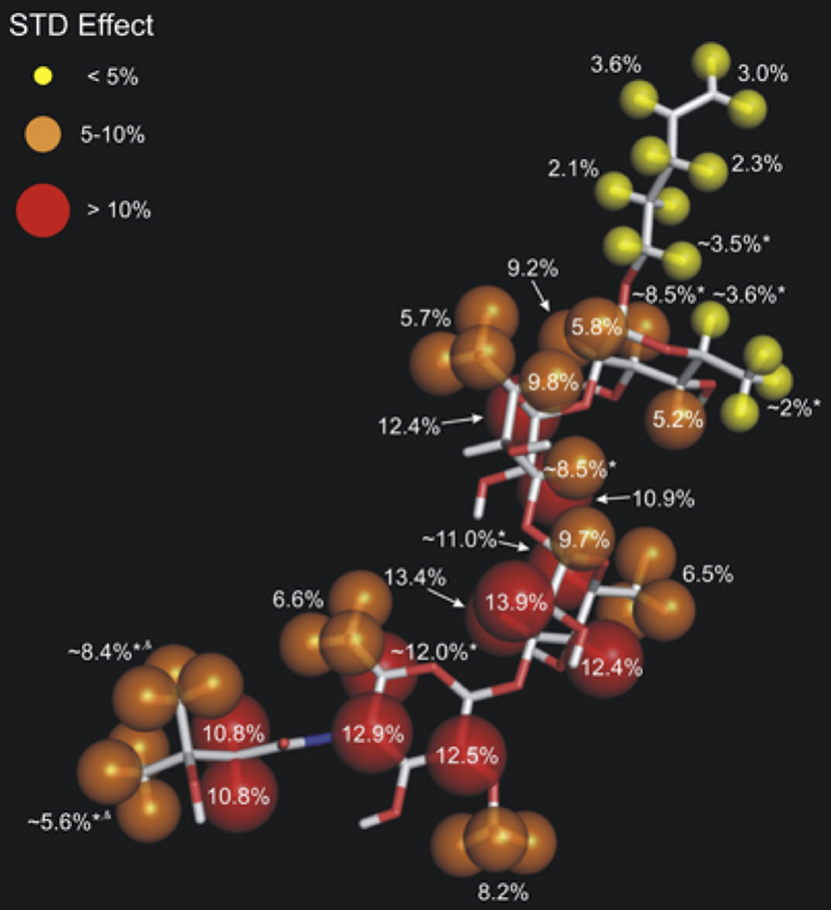
Epitope mapping of a synthetic tetrasaccharide resembling glycans on the surface of *Bacillus anthracis* and a monoclonal antibody raise against this tetrasaccharide epitope by STD NMR spectroscopy. STD effects are shown for individual protons of the tetrasaccharide and strong (>10%), medium (5–10%), and weak (<5%) STD effects are indicated by red, orange, and yellow spheres of decreasing size. Reprinted with permission from ref (121). Copyright 2010 American Chemical Society.

Quantification of the carbohydrate-antibody binding interactions was performed by SPR and compared to the qualitative glycan array screening results. SPR analysis also reveals interaction kinetics. STD NMR is particularly suited for characterizing binding differences within ligands to discriminate tightly bound domains from weakly bound domains without having to assign the resonances of the macromolecular receptor. However, slow kinetics results in very limited transfer of ligands from the antibody-bound state to the free state, and greatly affect the signal-to-noise ratio of STD NMR experiments. Strong STD effects indicate tight-binding sites. These interactions can be located throughout the entire glycan, as was the case for *B. anthracis* tetrasaccharide, with a cluster of tight-binding sites found within the anthrose-(β1–3)-rhamnose substructure (Figure 7). A combination of microarray profiling, SPR, and STD NMR allows for precise mapping of the molecular elements of glycan-antibody binding. The approach is a general tool that may ultimately help to elucidate the general principles of carbohydrate-antibody interactions, enabling guided structure-based design of a broad spectrum of carbohydrate-based antigens and therapeutics.^121^

#### 1.3.10 Opsonophagocytic Killing As a Test for Bactericidal Activity

Protective antibodies often function by opsonization and promote complement-mediated lysis of target pathogens.^53^ Therefore, typically sera from immunized mice or rabbits are tested for their opsonophagocytic potential in a standardized opsonophagocytosis assay (OPA).^122^ Human promyelocytic leukemia cells (HL-60) are differentiated into neutrophil-like cells before bacterial cells are incubated with test sera from naïve or immunized animals and then added to differentiated HL-60 cells in the presence of complement. After 1 h, the remaining viable bacterial cells are quantified by plating and subsequent counting of colonies (CFU-assay). Serum from immunized animals is expected to mediate dose-dependent killing of bacteria demonstrating that the conjugate vaccine elicits opsonophagocytic antibodies. Antibacterial serum antibody titers as well as opsonophagocytic activity are typically strongly dependent on the adjuvant. FA typically elicits higher antibody titers with higher killing-activity in the OPA assay compared to aluminum phosphate adjuvanted glycoconjugates.

#### 1.3.11 Animal Challenge Experiments to Compare Vaccinated and Control Groups

After establishing that a glycoconjugate is immunogenic, its immunoprotective properties against a bacterium of interest are evaluated in an appropriate animal model. The glycoconjugate is typically formulated with Alum as an adjuvant allowed for use in humans or is used unadjuvanted. Animals are typically immunized three times in two week intervals with 2–5 μg of glycoconjugate per immunization and antibody titers are measured. The naïve mice and those immunized with glycoconjugate are then infected with the bacterium of interest and clinical signs of disease are monitored. As a direct indicator of antibacterial defense in naïve and immunized mice, bacterial burdens at a certain time point are quantified to assess the protective effects of immunization on bacterial growth. Ideally, immunization with glycoconjugate reduces bacterial loads when compared to PBS-treated animals or those immunized with the glycoconjugate without adjuvant. Moreover, immunization should almost completely prevent bacteremia that is observed in vaccine-naïve control animals.

Induction of long-lived immunity is an important feature of successful vaccination. When mice are used as a model system, resting the animal for 90 days following the last booster shot before one additional dose is given tests the long-term response. Prior to the final injection, the antibody levels have typically dropped to very low levels but rapidly rise upon a boost. Good long-term effects of glycoconjugate immunization and a boostable immune response are important positive indicators for the preclinical and clinical development of carbohydrate-conjugate vaccine candidates.

## 2 Case Studies of Semi-Synthetic Glycoconjugate Vaccine Development

The conceptual path to identify a semisynthetic vaccine candidate for further preclinical and clinical development (section 1.3) has been followed for different pathogens and many different serotypes. In this section, specific lessons learned during the development of vaccine candidates will be discussed.

### 2.1 Synthetic Glycotope

Currently marketed vaccines based on isolated polysaccharides contain a myriad of glycotopes that are presented to the immune system. The initial focus of the development of semi- or fully synthetic glycoconjugate vaccines is the medicinal chemistry effort aimed at identifying a single protective epitope. Identification of such a minimal epitope provides the basis for further development but also constitutes the composition of matter intellectual property to protect the vaccine product. Based on insights concerning the RU of a bacterial CPS or a LPS structure, a host of questions must be answered experimentally: (i) What is the minimally required length of the glycotope, is one RU enough or are several needed? (ii) What frameshift is best to use, or in other words, which should be the terminal glycan? (iii) Are covalent modifications of the glycan backbone important? (iv) Are there any labile groups in the antigen that might be altered during the conjugation process to the carrier? Typically, multiple rounds of chemical synthesis of a host of oligosaccharides (see section 1.3.3) and subsequent immunological evaluation (see sections 1.3.4–1.3.8) are required.

#### 2.1.1 Glycotope Length

Since pathogens such as bacteria are surrounded by polysaccharides often containing hundreds of monosaccharides, the fundamental question for the development of synthetic oligosaccharide-based vaccines concerns the size of the glycan epitope required to induce a protective immune response in a living organism. At the outset of the work on synthetic carbohydrate vaccines it was not clear whether short oligosaccharides were sufficient to provide a protective immune response. Subsequently, pioneering work by Kamerling in the late 1980s and 1990s showed that short oligosaccharides are sufficient to induce a protective immune response.^28^ It is difficult to generalize how many RUs of a given CPS are required as the length of an RU may vary from mono- to hexasaccharides.

**Figure 8:**
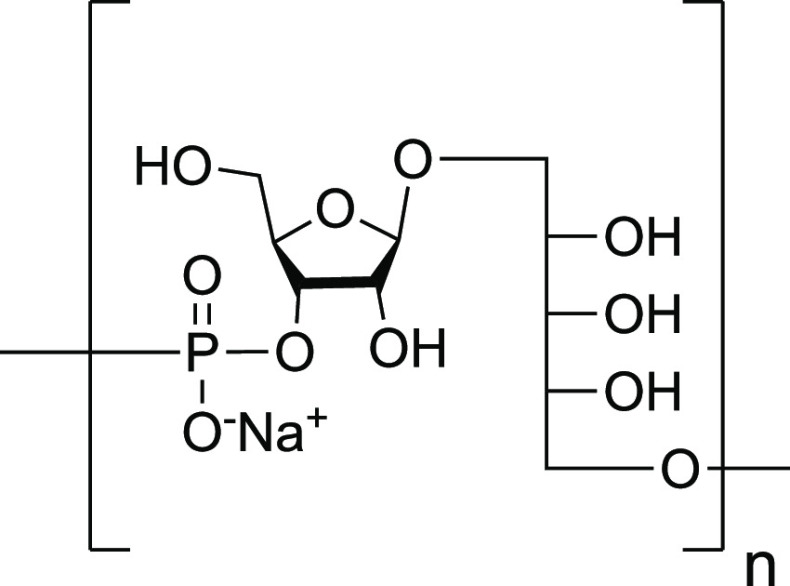
Structure of the *H. influenzae* type b CPS RU. n = number of repeating units.

*Haemophilus influenzae* type b (Hib) is an interesting case to study minimal glycotope length. The bacterium is covered by the PRP polymer (Figure 8). Licensed glycoconjugate vaccines are produced from PRP that is isolated from bacterial fermentation and is often size reduced.^15^

PRP served as a target for carbohydrate synthesis for 30 years.^123−126^ Semisynthetic glycoconjugates, containing a mixture of different length glycotopes produced by polymerization of the RU resulted in the first approved glycoconjugate vaccine containing a synthetic oligosaccharide hapten and TT now in routine use in Cuba (Quimi-Hib).^48^ Though clinically effective, the oligosaccharide component of Quimi-Hib is a mixture of oligosaccharides, six to eight RUs on average, obtained by polycondensation. Synthetic PRP oligosaccharide antigens of defined length were prepared using a disaccharide building block and elongation via H-phosphonate chemistry following a [2 + 2], [4 + 2], [6 + 2], and [8 + 2] synthesis strategy using orthogonal protecting groups.^120^ The synthetic PRP oligosaccharides, similar to natural Hib PRP, were immobilized on glycan arrays for antibody analysis and coupled to CRM197 for immunizations. Glycan array analyses revealed that synthetic PRP oligosaccharides present cross-reactive epitopes to antibodies raised against isolated PRP. Glycoconjugates of PRP oligosaccharides are immunogenic in a rabbit model (mice are a very poor model to study PRP immunogenicity), whereby tetrameric PRP is an excellent starting point for the design of a defined semisynthetic glycoconjugate Hib vaccine.^120^

**Figure 9:**
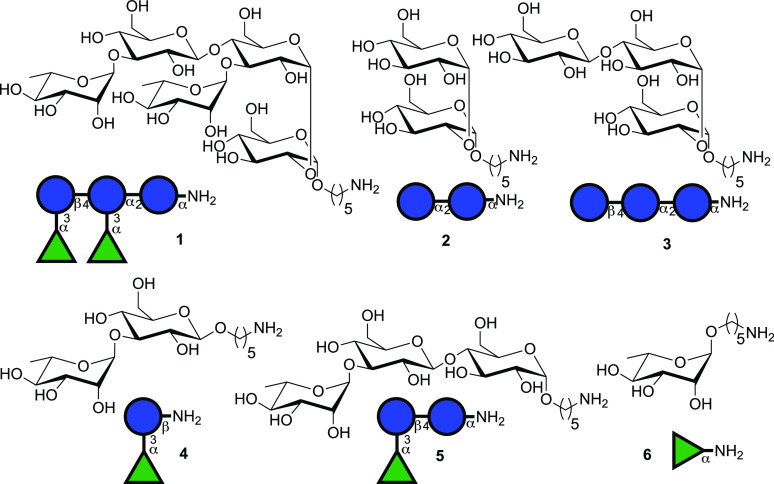
Synthetic PS-I pentasaccharide RU **1** and related glycan substructures **2**–**6**.

*Clostridium difficile* bacteria are covered with three different types of polysaccharides termed PS-I, PS-II and PS-III.^67,68^ Cell-surface polysaccharide PS-I consists of a pentasaccharide phosphate RU [ → 4)-α-Rhap-(1 → 3)-β-Glcp-(1 → 4)-[α-Rhap-(1 → 3)]-α-Glcp-(1 → 2)-α-Glcp-(1 → P] and has been identified on several *C. difficile* strains.^127^ PS-I related glycans are poorly expressed under culture conditions.^68^ A series of PS-I related oligosaccharides was prepared to identify the minimally protective epitope (Figure 9).^104^ A pentasaccharide-CRM197 conjugate was immunologically evaluated. The glycoconjugate proved immunogenic in mice and immunoglobulin class-switching and affinity maturation were observed. Glycan array-assisted epitope mapping revealed the Rha-(1 → 3)-Glc disaccharide to be the minimal epitope of the pentasaccharide. CRM197 glycoconjugates of pentasaccharide 1 as well as disaccharide 4 are promising vaccine candidates to protect from *C. difficile* infection; these are currently in further preclinical evaluation.^119^

**Figure 10:**
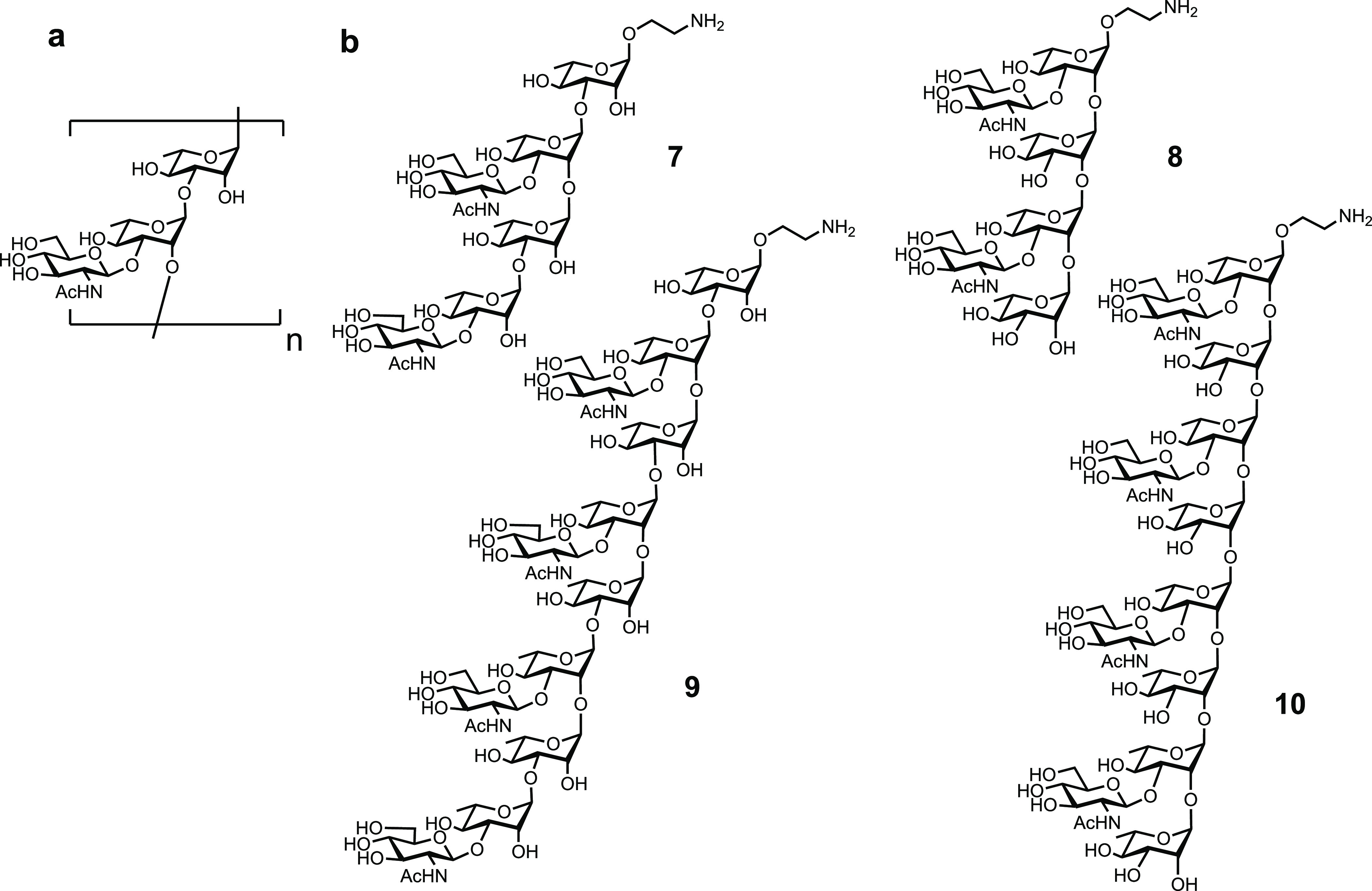
(a) Structure of the cell-wall polysaccharide RU of Group A Streptococcus and (b) related synthetic oligosaccharides (**7**–**10**) utilized for immunological investigations. n = number of repeating units.

Group A Streptococcus (GAS) bacteria contain a surface polysaccharide consisting of repeating [ → 3)α-L-Rhap(1 → 2)[β-D-GlcpNAc(1 → 3)]α-L-Rhap(1-]n units (Figure 10). The helical GAS polysaccharide is conserved and abundantly expressed on most GAS serotypes (Figure 10a). Purified GAS-polysaccharide conjugated to tetanus toxoid carrier elicited a protective immune response in a mouse challenge model.^128^ A hexamer of two RUs was reported as core antigenic determinant believed to be recognized by the human anti-GAS humoral immune response.^129^ A series of synthetic oligosaccharides (**7**-**10**) was used to study the effect of carbohydrate length and composition on immunogenicity.^130^ Dodecasaccharide–CRM197 glycoconjugates (Figure 10b) exposing an immunodominant GlcNAc sugar on the nonreducing terminus elicited specific IgG titers in mice, comparable to those induced by CRM197-GAS-PS, while shorter oligosaccharides (**7**-**9**) were less efficacious. Thus, the minimal length epitope was determined to be a 12-mer. Immunoprotection studies in a mouse challenge model and opsonophagocytosis in vitro assays with specific rabbit antisera demonstrated that synthetic conjugate vaccine candidates have similar efficacy to conjugates of isolated GAS-polysaccharide.^131,132^

#### 2.1.2 Glycotope Frameshifts

Vaccines based on isolated polysaccharides contain long chains of many RUs such that the nonreducing terminus constitutes only a small part of the overall glycan. In shorter oligosaccharides, the nonreducing end sugar makes up a much larger portion of the total glycan. It is this terminal portion of the glycan that is believed to interact predominantly with the mammalian immune system. The nonreducing terminus may play a major role in immunogenicity albeit not antigenicity and protection. When just a single RU is the minimal glycotope, the terminal residue may be particularly important. A RU of four sugars may be depicted in generic terms as ABCD, BCDA, CDAB or DABC, depending on the frameshift, and it must be determined by chemical assembly and immunological testing which is most effective in inducing a protective immune response.

**Figure 11:**
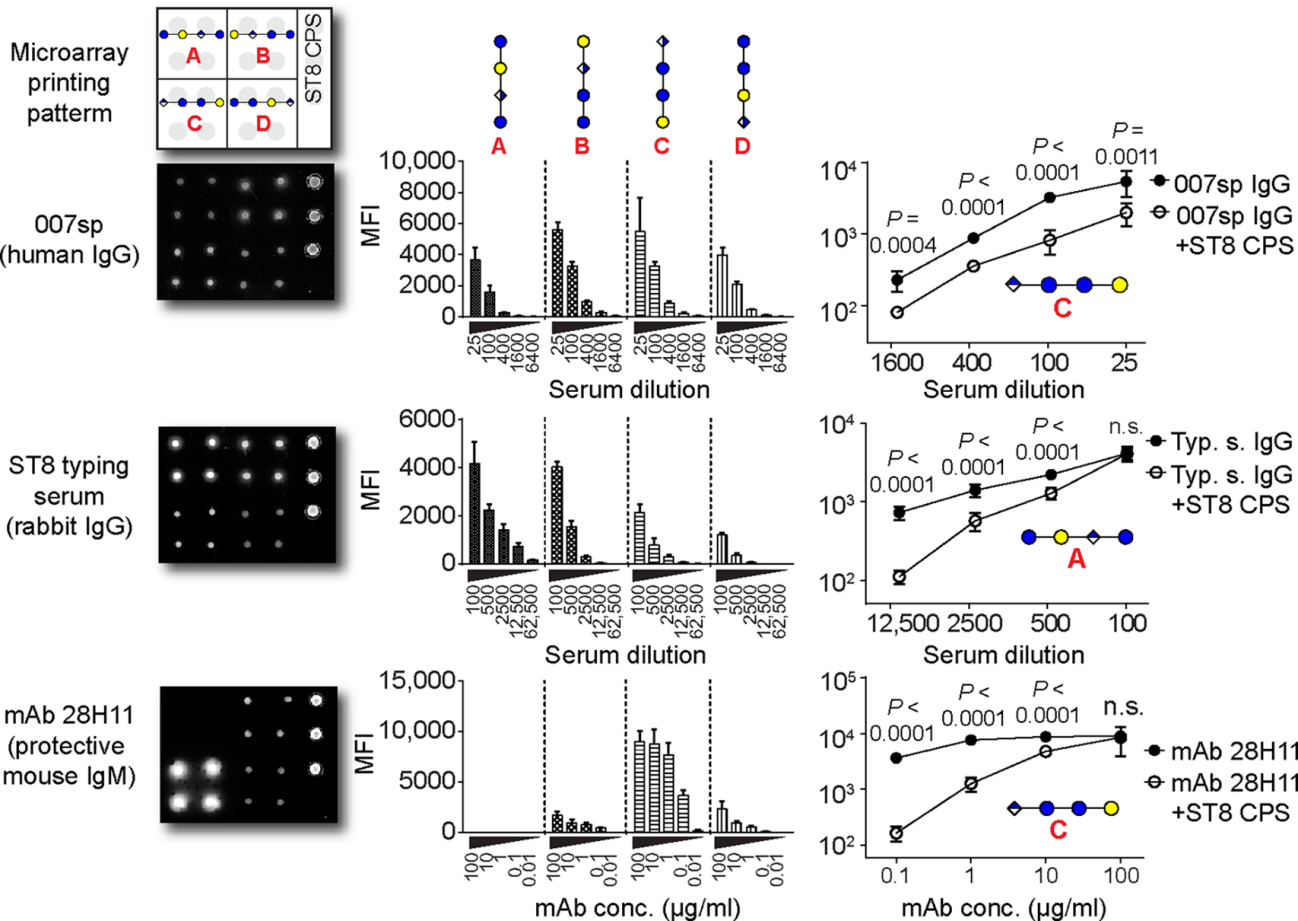
Differential immune recognition of synthetic SP-8 CPS frameshift oligosaccharides **A**–**D**. Glycan microarray analysis of pooled sera from Pneumovax 23-vaccinated humans (International Reference Serum 007sp), rabbit SP-8 typing serum and a protective murine mAb 28H11 at different concentrations. Sera were preadsorbed with pneumococcal C-polysaccharide before application. Histograms show mean + SD of eight spots. Inhibition of antibody binding by preadsorption with native ST8 CPS (10 μg/mL). Statistical analysis (one-tailed, unpaired *t* test with Welch’s correction) of eight spots was performed of one out of at least two independent experiments. Asterisks indicate P values: n.s. not significant; **P *<* 0.005; ***P *<* 0.001; ****P *<* 0.0001. Bars depict mean + SD. MFI, mean fluorescence intensity. Reprinted with permission from ref (133). Copyright 2017, American Association for the Advancement of Science.

The search for the optimal frameshift was explored in the context of oligosaccharide vaccine development for *S. pneumoniae* serotype 8 (SP-8). Four tetrasaccharide frameshifts of the native SP-8 polysaccharide were prepared by automated glycan assembly. The availability of all SP-8 frameshift oligosaccharides was crucial to gain insight into the presence of protective glycotopes. Yet, a more comprehensive glycan collection was required to exclude nonprotective glycotopes. In-depth characterization of protective FS C-directed mAbs revealed a trisaccharide glycotope that was weakly immunogenic in mice, but induced a robust antibacterial immune response as a vaccine antigen in rabbits (Figure 11).^133^

#### 2.1.3 Sugar Modifications

Carbohydrate modifications can strongly impact glycan immunogenicity. CPS of bacterial pathogens are frequently equipped with acetates, pyruvates and other appendages. Since such modifications are not found on human glycans, they are believed to be immunogenic and potentially antigenic and therefore important components of neoglycoconjugate vaccines.

##### 2.1.3.1 Pyruvate: *S. pneumoniae* Serotype 4

**Figure 12:**
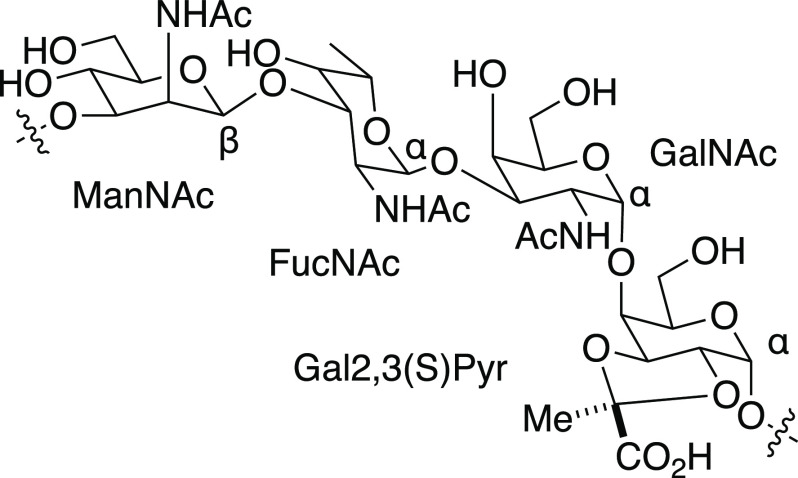
RU of *S. pneumoniae* serotype 4 CPS.

A pyruvate molecule can be placed across the (4, 6)-, (3, 4)-, or (2, 3)-positions. The CPS of the prevalent *S. pneumoniae* serotype 4 (SP-4) is composed of tetrasaccharide RUs and is included in existing pneumococcal vaccines. A trans-(2, 3)-linked pyruvate is present on the SP-4 CPS (Figure 12). The structural antigenic determinants that are essential for protective immunity, including the role of the rare and labile cyclic *trans*-(2,3) pyruvate ketal modification, were largely unknown. Key antigenic determinants of SP-4 CPS were determined with the help of pyruvated and nonpyruvated synthetic RU glycans.^134^ Glycan arrays revealed which oligosaccharide antigens were recognized by antibodies in the human reference serum. Depyruvated SP-4 oligosaccharides were highly immunogenic, but the resulting antiglycan antibodies showed only limited binding to the natural CPS present on the bacterial surface. Glycan array and surface plasmon resonance analysis of murine polyclonal serum antibodies as well as monoclonal antibodies showed that terminal sugars are important in directing the immune responses. The pyruvate modification was a key motif in designing minimal synthetic carbohydrate vaccines against SP-4.^135^

##### 2.1.3.2 3-Hydroxy-3-methylbutanamide: *Bacillus anthracis*

**Figure 13:**
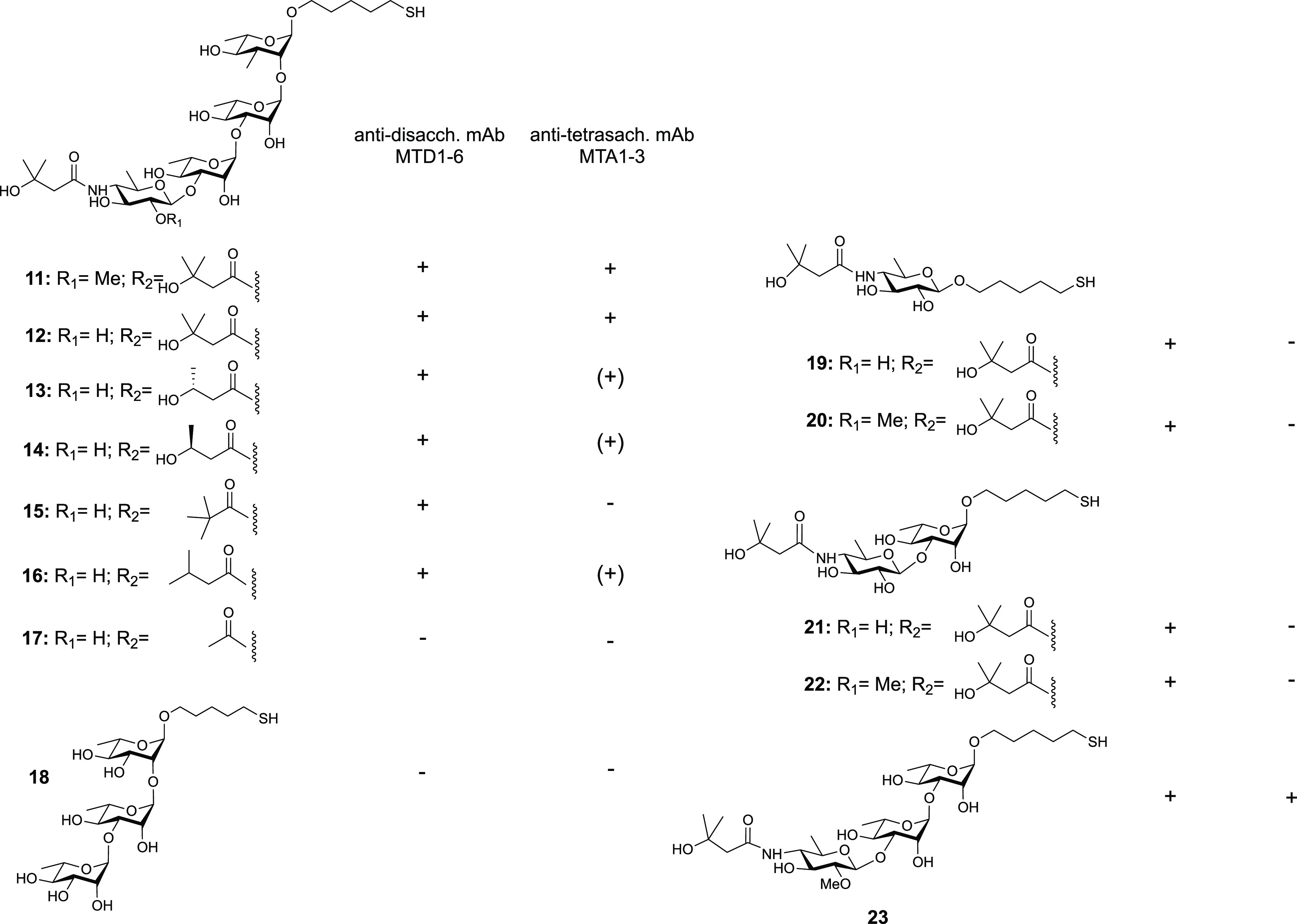
Synthetic glycans (**11**–**23**) related to the *B. anthracis* cell surface tetrasaccharide of BclA used for antibody mapping by microarray screening. Microarray analysis demonstrates the cross-reactivity of monoclonal antibodies generated against anthrose-rhamnose disaccharide **22** (MTD1-MTD6) and tetrasaccharides **11** and **12** (MTA1-MTA3).

A tetrasaccharide found on the glycoprotein BclA on the *B. anthracis* cell surface contains three rhamnose residues and an unusual terminal sugar, 2-O-methyl-4-(3-hydroxy-3-methylbutanamido)-4,6-dideoxy-d-glucopyranose, named anthrose. The 3-hydroxy-3-methylbutanamido side chain of anthrose was suspected of being immunodominant in this antigen. Synthetic glycans were screened for their ability to bind antidisaccharide or antitetrasaccharide mAbs (see also Section 1.3.9, Figure 7).^121^ A drastic truncation of the chain, produced by reducing 3-hydroxy-3-methylbutyrate to acetate (compound **17**, Figure 13) resulted in a structure that was not recognized by any mAb. However, deleting a methyl group within the side chain (compounds **13** and **14**, Figure 13), reduced binding to some Abs but not to others.^121^ Similarly, placement of a trimethylacetyl moiety (compound **15**, Figure 13), or deletion of a 3-hydroxyl group (compound **16**, Figure 13) only affected some mAbs.^121^

##### 2.1.3.3 Acetates: PNAG

Poly-*N*-acetylglucosamine (PNAG) is a surface polysaccharide produced by a broad range of common pathogens and consists of a β-(1–6)-linked polymer of *N*-acetyl-d-glucosamine (GlcNAc) whereby some of the amino groups lack the acetate substituent. PNAG is produced by *Staphylococci aureus* and *epidermidis*,^136^*E. coli*, *Bordetella pertussis Acinetobacter spp.*, and *Yersinia pestis*. Conjugate vaccines containing highly but not completely deacylated forms of PNAG are effective at providing protective immunity in animals. The lack of definition of the chemical composition and the variability of the vaccine formulation made it difficult for prior studies to draw final conclusions concerning the vaccine candidate. The position and spacing of deacetylated GlcNac units was not clear. In order to determine the relative numbers of glucosamine units and their spacing penta- or nonasaccharide acetylated oligoglucosamines were compared to the same length molecules without any acetylation at the amine groups. The fully acetylated oligosaccharides elicited high titers of nonopsonic antibodies in mice, whereas the oligosaccharides containing no acetates elicited no highly active opsonic antibodies in mice and rabbits. Clearly, the presence of acetate groups is important for immunogenicity and antigenicity.^137^ Details concerning the impact of acetylation content and spacing have yet to be revealed.

#### 2.1.4 Unstable Epitopes

Modifications of cell-surface glycans are quite frequently found in bacteria and other pathogens. These appendages are often key to immunogenicity and antigenicity (vide supra). Undesired modifications of glycans can, however, also arise during the purification of a glycan from biological sources or during its conjugation to a carrier protein. The reaction of such unstable epitopes may result in the formation of neo-epitopes and/or the destruction of epitopes that are required for molecular recognition. Antibody responses to the novel epitopes may lead to problems such as autoimmunity or to loss of activity when key epitopes are no longer recognized.^138^ The precise modification of glycans can vary as a result of their isolation process, which defines the product. Production problems due to product variations increase manufacturing costs and uncertainties.

##### 2.1.4.1 Ketone: *S. pneumoniae* Serotype 5

**Figure 14:**
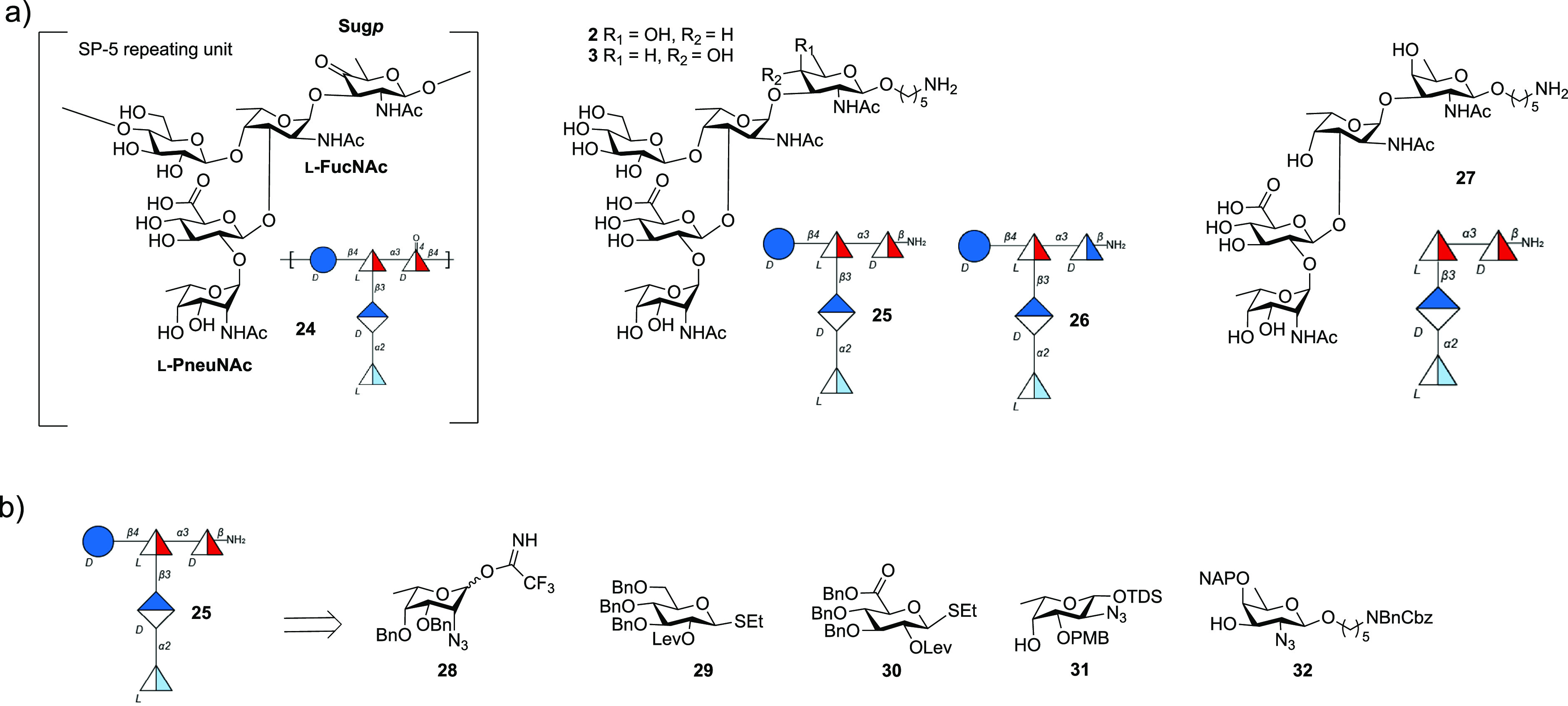
(a) Line and symbol structures of SP-5 CPS RU **24** and synthetic antigen targets (**25**–**27**); (b) retrosynthetic analysis of target **25**.

Although SP-5 is the fifth most prevalent serotype, causing invasive pneumococcal disease among young children globally, current glycoconjugate vaccines are not fully efficacious in preventing SP-5 infections.^16^ A change in the CPS glycan structure during antigen isolation and purification such that the SP-5 antigen no longer sufficiently resembles the native CPS was suspected of compromising vaccine efficacy.^139^ The SP-5 branched pentasaccharide RU structure (Figure 14)^138,140^ contains two rare deoxyamino sugars, the ketoamino sugar 2-acetamido-2,6-dideoxy-d-xylose-hexos-4-ulose (Sugp) and *N*-acetyl-l-pneumosamine (l-PneuNAc).

Marketed glycoconjugate vaccines are manufactured from either native or depolymerized CPS that are, following isolation, typically coupled to a carrier protein via reductive amination. The keto group present in the rare sugar Sugp is partially or fully reduced to form a mixture of SP-5 CPS components and degrades during glycoconjugate production and leads to manufacturing issues and decreased immunogenicity.

Defined synthetic antigens resembling the SP-5 CPS RU **24** provided valuable insights into how changes to the natural SP-5 CPS may influence antigen stability and immunogenicity.^141^ Chemical synthesis provided access to the natural keto containing RU **24**, as well as reduced form oligosaccharides **25**–**27** (Figure 14) to probe general aspects of vaccine design relating to the effect of branching, length and the role of unique sugars like L-PneuNAc and Sugp. CRM197-**25** conjugate formulated with the adjuvant aluminum hydroxide stimulated more cross-reactive antibodies than CRM197-**27** against the native SP-5 CPS following injection into rabbits. The d-glucose residue, present in **25**, but not in **27**, proved vitally important for antibody recognition and cross-reactivity. The SP-5 epitope responsible for specificity (l-PneuNAc, d-Glc) differs from the reducing end sugar (d-FucNAc). The branched pentasaccharide **25** is more immunogenic and a better mimic of the native ST-5 CPS as conjugate CRM197-**25** produced CPS specific cross-reactive antibody titers, in contrast to linear tetrasaccharide conjugate CRM197-**27**. Glycan array analyses of human reference sera and immunization experiments in rabbits identified the rare aminosugar l-PneuNAc, as well as branching as key to antibody recognition and avidity. Oligosaccharide **25** containing a secondary alcohol in place of the labile ketone in native SP-5 CPS **24** resulted in improved antibody titers and opsonic activity when compared to Prevnar13 that contains natural SP-5 CPS.^141^ The medicinal chemistry approach allowed for the identification of key epitopes and the replacement of nonessential, labile entities that create production problems with closely related, stable functional groups.

### 2.2 Linker Chemistry to Create Glycoconjugates

A small but important piece of the glycoconjugates used as vaccines is the linker that combines the glycan (synthetic or isolated) with the carrier (typically a protein). While the coupling between glycan and carrier has to occur selectively and efficiently, the resulting linker should be nonimmunogenic. In traditional glycocconjugate vaccines where isolated glycans are used, reactive groups present on the polysaccharide are used for attachment and a host of different points of attachment can be envisioned. When synthetic oligosaccharides are used, a unique functional group can be placed for single point attachment. Typically, the reducing-end of the glycan is equipped with a linker that contains either an amine or a thiol group. This functional group can then be selectively coupled to a linker and to the amine side chains on the carrier protein. A myriad of different conjugation methods for synthetic oligosaccharides exist and have been extensively reviewed.^142^

#### 2.2.1 Attachment via a Terminal Amine

**Figure 15:**
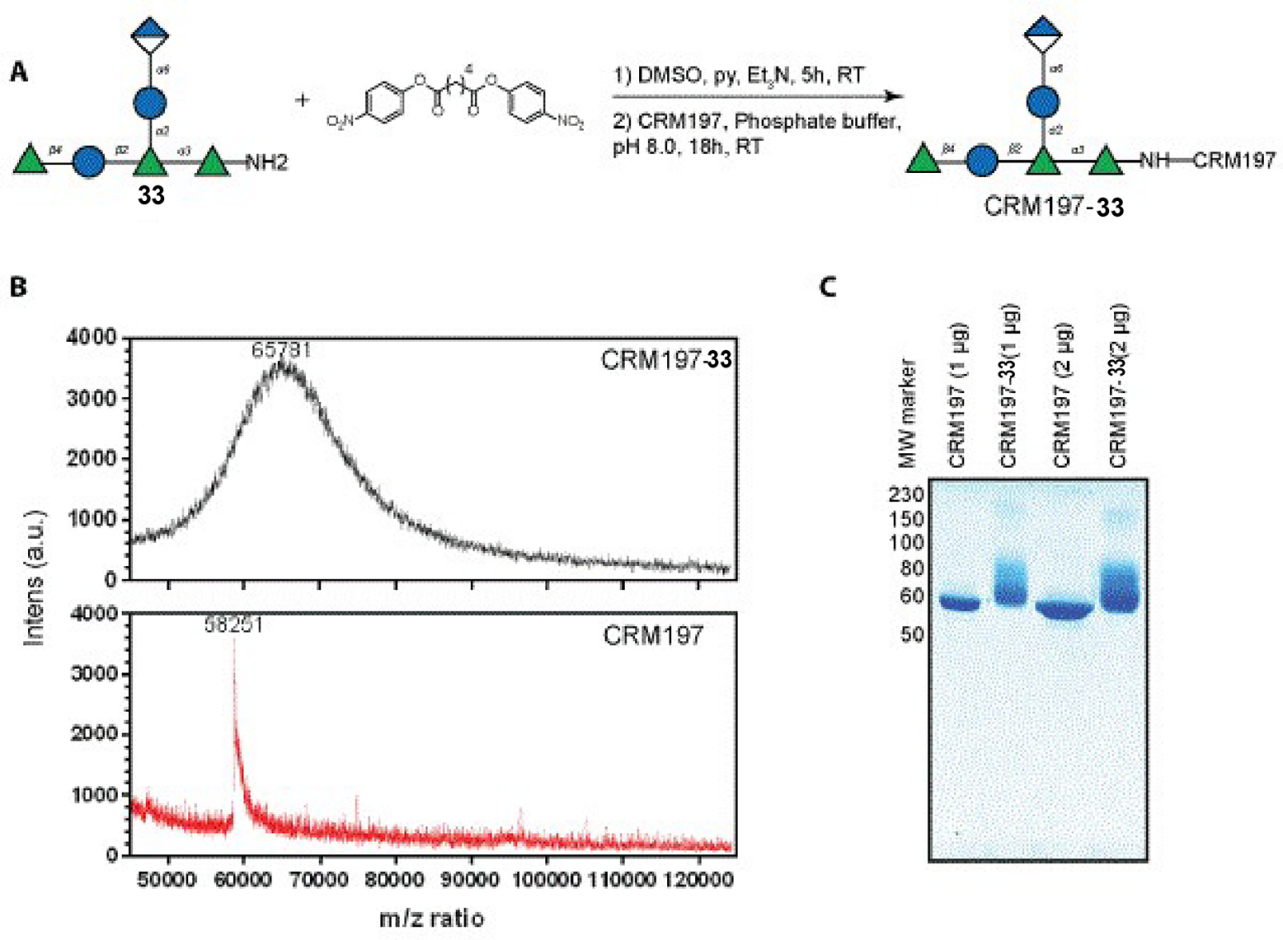
Preparation and characterization of a *S. pneumoniae* serotype 2 (SP-2) CRM197 neoglycoconjugate. (A) Schematic representation of CRM197-**33** conjugate. Hexasaccharide **33** was covalently coupled with CRM197 using *p*-nitrophenyladipate ester as a coupling reagent. (B) MALDI-TOF analysis was used to determine the average molecular weight of the conjugate; CRM197 was used as a standard. (C) The CRM197-**33** conjugate and CRM197 were resolved with 10% SDS-PAGE and stained with PageBlue protein staining solution. The molecular weight marker is indicated on the left.

Many site-selective methods for the attachment of different molecules to proteins have been developed, recently fueled by programs developing antibody-drug conjugates. *p*-Nitrophenyladipate ester has proven to be an effective coupling reagent to conjugate glycans to proteins (Figure 15).^142−150^ The success of the coupling reaction is assessed by MALDI-TOF mass spectrometry and confirmed by SDS-PAGE.

#### 2.2.2 Attachment via Terminal Thiols

In most cases, synthetic glycans are equipped with amine-containing linkers that are used to form adducts with suitable electrophiles. This conjugation method cannot be used when amines are present in the glycan antigen to be conjugated. A small, but important class of bacterial polysaccharides harbor RUs with zwitterionic charge motifs that typically also contain amine groups. These zwitterionic polysaccharides (ZPSs) exhibit unique immunomodulatory activity and are commonly associated with commensalism.^151−156^ ZPSs are the first carbohydrate-only antigens to induce a T cell-dependent immune response through a MHC class II dependent pathway.^157−161^

**Figure 16:**
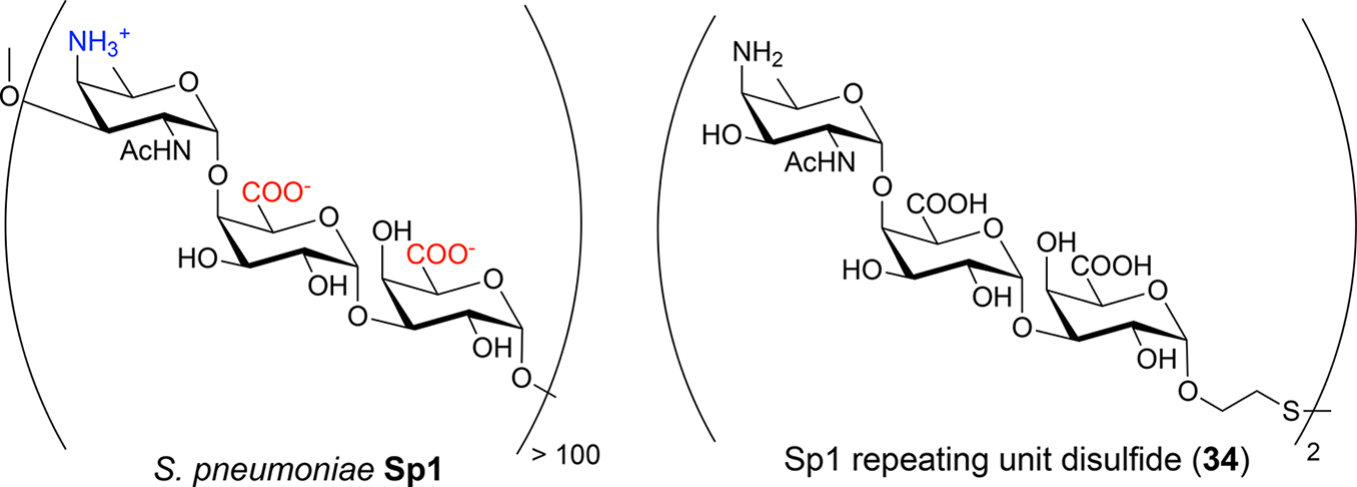
*S. pneumoniae* serotype 1 (SP-1) is a natural zwitterionic polysaccharide (ZPS) and the synthetic oligosaccharide **34** resembling SP-1 is conjugated to the carrier protein via a terminal thiol.

Thiol-linked glycans have seen limited use for oligosaccharide conjugation since the thiol moiety is usually introduced at the very end of a synthesis due to incompatibilities with oxidation reactions in oligosaccharide assembly. The RU of *S. pneumoniae* SP-1 is a ZPS trisaccharide consisting of two d-galacturonic acid moieties and the rare aminosugar 2-acetamido-4-amino-2,4,6-trideoxy-d-galactose (D-AAT).^162−164^ Synthetic SP-1 glycan **34** equipped with thiol linkers enabled the conjugation to glycan arrays and CRM197 carrier protein (Figure 16).

Marketed glycoconjugate vaccines induce low levels of functional antibodies against the highly invasive serotype 1 (SP-1), presumably due to obscuring of protective epitopes during chemical conjugation to carrier proteins. Synthetic oligosaccharide antigens can incorporate linkers for site-selective protein conjugation while keeping protective epitopes intact. An efficacious semisynthetic SP-1 glycoconjugate vaccine candidate was identified using a panel of synthetic oligosaccharides that revealed a critical role of the rare aminosugar 2-acetamido-4-amino-2,4,6-trideoxy-d-galactose (d-AAT). A SP-1 trisaccharide conjugate carrying D-AAT at the nonreducing end induced a strong antibacterial immune response in rabbits and outperformed the SP-1 component of the multivalent blockbuster vaccine Prevnar 13.^165^

### 2.3 Carriers

Carbohydrates are T-cell independent antigens and fail to induce a protective immune response in small children. Therefore, the combination of a carbohydrate B-cell epitope with a carrier that includes a T-cell epitope is essential to induce a protective, long-lasting immune response in patients. To date protein carriers are used exclusively in marketed vaccines but a host of carriers have been explored as potential components of next-generation vaccines.

#### 2.3.1 Glycoprotein Carriers

Carrier proteins are isolated following expression in bioreactors, using well-characterized bacterial strains.^107^ A solution of pure protein is obtained after several chromatography steps and sterile filtration.^16^ Carrier proteins such as TT, DT, and CRM197 are well-established in carbohydrate-based vaccines as they provide the needed T-cell help for immunological memory. However, the side effects observed due to the high immunogenicity of commonly used carriers have prompted the search for alternatives. In addition, finding nonprotein carrier platforms may alleviate the need of a cold chain for transportation and storage of vaccines. Not having to maintain a cold chain would tremendously lower the cost per dose of vaccine.^166^

#### 2.3.2 Glycolipid Carriers

Efforts to exploit alternative methods for antigen presentation aim to utilize the potent helper functions of invariant natural killer T (iNKT) cells to drive the production of anticarbohydrate antibodies that protect against bacterial infection. Activation of iNKT cells is mediated by self-and exogenous lipid antigens presented by the nonpolymorphic antigen-presenting molecule CD1d, which is expressed by both lymphoid lineages (including all B cells) and nonlymphoid lineages. iNKT cells are “preprimed” in vivo, exhibit a memory phenotype, and are capable of rapid cytokine release upon antigen activation. While iNKT cells typically comprise less than 0.1% of circulating T cells in humans, their number exceeds that of naïve T cells specific for individual peptide antigens. Rather than responding to peptides, iNKT cells are activated by a potent lipid agonist known as α-galactosylceramide (α-GalCer)^167^ that efficiently stimulates iNKT cells at very low doses. α-GalCer has been used as an adjuvant in murine models of disease,^168−171^ and in human clinical trials.^172^ Covalent conjugation of α-GalCer to pneumococcal polysaccharides promote iNKT cell help for B cell production of the respective anticarbohydrate antibodies.^173^

**Figure 17:**
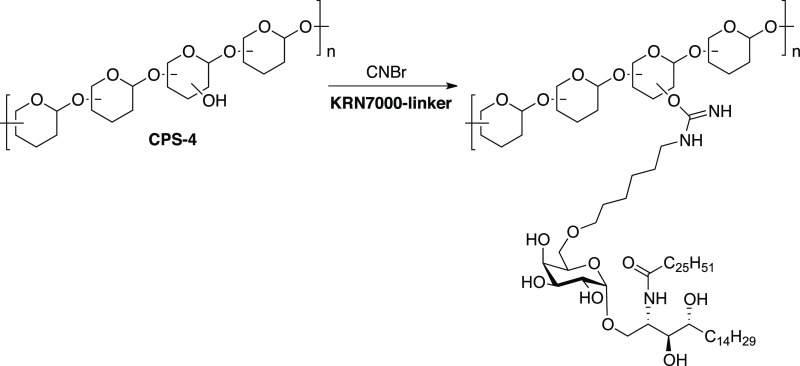
Coupling of *S. pneumoniae* serotype 4 CPS to the iNKT cell agonist α-GalCer.

To create a molecule that could provide iNKT cell help for B cell production of *S. pneumoniae* polysaccharide-specific antibodies, the *S. pneumoniae* serotype 4 CPS (Figure 12) was coupled to the iNKT cell agonist α-GalCer (Figure 17).^174^ The CPS-αGC compound was constructed such that B cell recognition of the CPS moiety was preserved to allow efficient uptake of the conjugate vaccine by B cells. Minimally destructive conjugation techniques that preserve the CPS epitopes were used as a linker that can be processed within B cell lysosomes to release the α-GalCer moiety to form immunogenic complexes with CD1d. An amine moiety was placed at the C6 carbon of the α-GalCer galactose so that lipid immunogenicity was not disrupted.^175^ This modified α-GalCer molecule was coupled to CPS using the widely used activator cyanogen bromide as the coupling reagent.

Vaccination with CPS-αGC induced the generation of high-affinity, carbohydrate-specific protective antibodies, the induction of carbohydrate-specific memory B cells, and conferred full protection against *S. pneumoniae* infection three months after the last boost. The high titers of IgG antibodies detected in sera from vaccinated mice, together with the isolation of mAbs specific for pneumococcal polysaccharide, demonstrated that glycoconjugate vaccination efficiently activates carbohydrate-specific B cells. This approach stimulated polysaccharide-specific B cells to switch to IgG production and undergo maturation into long-lived memory B cells. Critically, mice immunized with the novel vaccine exhibited long-lasting protection against *S. pneumoniae*.^174^

#### 2.3.3 Multivalent Carbohydrate Display

Many new carrier strategies focus on a multivalent hapten presentation. Encouraging results in vaccination experiments have been obtained with oligosaccharide-bound virosomes, liposomes, and gold nanoparticles (GNP).^113−115^ The fate of fully synthetic carriers, such as GNP inside an organism is often unclear. Thus, both in vivo toxicity and clearance pathways have to be accurately assessed for these materials.

Self-assembling two-component peptide fibers exposing Muc1 epitopes were created from peptide Q11 and Muc1 glycopeptides that in turn were assembled by solid phase peptide synthesis.^176^ The constructs showed adjuvant-like activity by the solid aggregates, but the high antibody titers are simply induced by the multivalent hapten display to B cells.^177^

The immunomodulatory properties of zwitterionic polysaccharides (see section 2.2.2) have been exploited in vaccine design. *Bacteroides fragilis* PSA1, the best studied ZPS representative, has been used as a carrier for the Tn antigen to produce an all-carbohydrate cancer vaccine candidate. The Tn-PSA1 conjugate was found to elicit a specific anti-Tn IgG response in mice without the need of an adjuvant.^178^ Antisera from these mice bound to cancer cells slightly better than antisera raised against unmodified PSA1.^179^ Vaccines created exclusively from carbohydrates are attractive since they do not require maintenance a cold chain.

## 3 Glycoconjugate Vaccine Candidates from Isolated and Synthetic Glycans

Almost all currently marketed glycocconjugate vaccines are based on isolated carbohydrates with the exception of Quimihib, a Cuban semisynthetic Hib vaccine prepared by chemical polymerization of a chemically derived building block. With fast and reliable chemical methods now available to define and immunologically test minimal glycotopes, the methodology to establish semisynthetic vaccines is available and has been employed to identify vaccine candidates for single bacterial serotypes that can be coformulated to expand the protection of already existing vaccines or to create novel multivalent vaccines.

### 3.1 Synthetic Glycoconjugate Vaccine Candidates for Bacterial Serotypes Without Vaccines

Many deadly pathogens carry unique glycan structures on their surfaces that may serve as a target for glycoconjugate vaccine development. Since in some cases, the pathogen is difficult to culture and in others the isolation of the polysaccharides in pure form is extremely difficult or completely impossible, the medicinal chemistry approach offers an attractive alternative. In this section, candidate vaccines for pathogens or serotypes where no vaccine is currently available are described.

#### 3.1.1 *S. pneumoniae*

Licensed polysaccharide conjugate vaccines cover currently only 13 of the over 90 serotypes of *S. pneumoniae* such that nonvaccine serotypes are a major obstacle to the effective control of invasive pneumococcal disease.

##### 3.1.1.1 SP-2

Serotype 2 is such a nonvaccine serotype that is the main cause of IPD in many countries in Asia and Middle America. To prevent IPDs caused by SP-2, the identification of an effective SP-2 neoglycoconjugate vaccine candidate by the medicinal chemistry approach was pursued. Glycan microarrays containing a series of synthetic glycans resembling portions of the SP-2 CPS RU were used to screen human and rabbit sera and identify epitope hits.^180^ Synthetic hexasaccharide **33** resembling one RU of SP-2 CPS emerged as a hit from the glycan array screens (Figure 15). Vaccination with neoglycoconjugates consisting of hexasaccharide **33** coupled to carrier protein CRM-197 was found to stimulate a T-cell dependent B-cell response that induced CPS specific opsonic antibodies in mice, resulting in killing of encapsulated bacteria by phagocytic activity. Subcutaneous immunization with neoglycoconjugate protected mice from intranasal challenge with a highly virulent SP-2 strain by reducing the bacterial load in lung tissue and blood.^180^

##### 3.1.1.2 SP-8

Highly virulent SP-8 causes frequent outbreaks of invasive disease. Even though SP-8 is part of the polysaccharide vaccine Pneumovax 23, it is not included in conjugate vaccines such as Prevnar 13. Many clinical SP-8 isolates are broadly resistant to antibiotics such that vaccination to prevent rather than fight SP-8 infections is advisible. The combination of AGA, glycan microarrays, and mAb reverse engineering produced semisynthetic glycoconjugate vaccines against SP-8 that were combined with Prevnar 13 to form experimental 14-valent vaccines.^181^

##### 3.1.1.3 SP-12F

**Figure 18:**
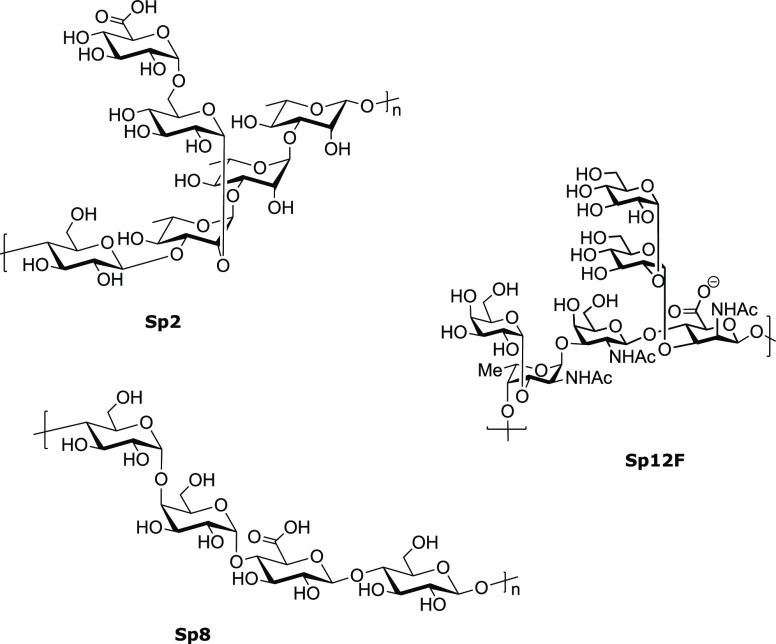
Structures of the *S. pneumoniae* serotypes SP-2, SP-8, and SP-12F capsular polysaccharide RU.^17^

The licensed polysaccharide vaccine Pneumovax 23 contains serotype 12F but is not efficacious in young children or elderly people, those at highest risk. The carbohydrate conjugate vaccines Prevanar13 and Synflorix do not contain SP-12F. Together, SP-12A and SP-12F account for more than 4% of pneumococcal disease^182^ and 12F (Figure 18) dominates with 85%.^183^ To identify a glycotope that can be incorporated into a next-generation glycoconjugate vaccine, the SP-12F hexasaccharide repeat unit (Figure 18) served as a first step toward a detailed immunological analysis.^184^

#### 3.1.2 *K. pneumoniae*

**Figure 19:**
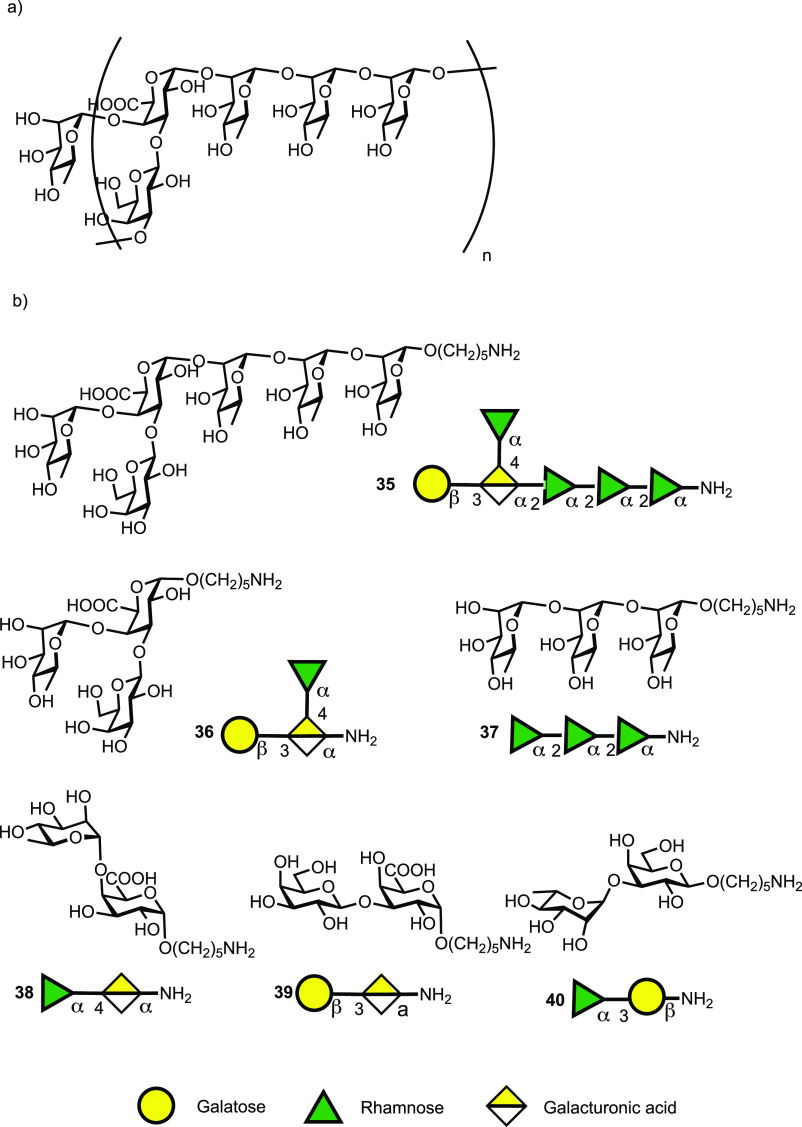
(a) Hexasaccharide RU from two CR-Kp clones isolated during a 2011 hospital outbreak; (b) synthetic hexasaccharide **35** and substructures **36**–**40** used for immunological studies.

Infections caused by carpabenem-resistant *Klebsiella pneumoniae* (CR-Kp) are very problematic hospital acquired infections, with a 50% average survival rate. At a time when antibiotics are becoming less effective, no vaccines to protect from this severe bacterial infection exist. The CPS surrounding CR-Kp is an attractive starting point to identify vaccine antigens. A synthesis of the CR-Kp hexasaccharide RU α-L-Rha-(1 → 4)-[β-D-Gal-(1 → 3)]-α-D-GalA-(1 → 2)-α-L-Rha-(1 → 2)-α-L-Rha-(1 → 2)-α-L-Rha and related sequences followed by glycan array studies identified the hexasaccharide as a starting point for the development of a synthetic glycoconjugate vaccine.^185^ Subcutaneous immunization with synthetic hexasaccharide-CRM197 conjugate resulted in high titers of cross-reactive antibodies against CR-Kp CPS in mice and rabbits. Whole cell ELISA revealed surface staining of CR-Kp strains. Anti-CRM197-**35** conjugate antibodies were found to promote phagocytosis in opsonophagocytic killing assays. The semisynthetic glycoconjugate is a lead for the development of a vaccine against a rapidly progressing, deadly bacterium (Figure 19).^185^

#### 3.1.3 *C. difficile*

**Figure 20:**
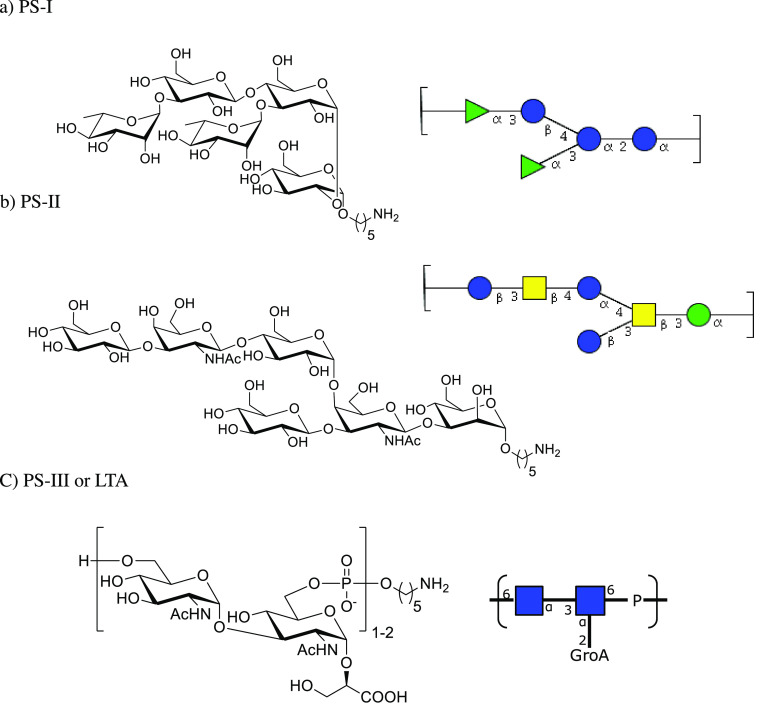
Synthetic oligosaccharide antigens resembling the polysaccharides on the surface of *C. difficile* bacteria that were used as basis for the development of glycoconjugate vaccine candidates.

The hospital acquired infection is currently treated with the antibiotics that contribute to disease recurrence in about 20% of patients by disrupting the gut microbiota. As antibiotics reach their limits, anti-*C. difficile* vaccine candidates have been pursued. Currently, three candidates are clinically investigated that induce antitoxin immunity, but do not prevent bacterial colonization.^186^ Vaccines targeting the bacterial surface, in contrast, could reduce the human reservoir and limit the spread of this emerging pathogen.^187^*C. difficile* surface-exposed polysaccharides, PS-I, PS-II, and PS-III, that are essential for bacterial survival and virulence^188^ emerged as targets for colonization-preventing vaccines.^67,68^ Glycoconjugates of isolated PS-II and PS-III were immunogenic in small animals.^189,190^ The production of clostridial polysaccharides is challenging due to their weak and inconsistent expression in bacterial culture.^191,192^ Synthetic PS-I,^193^ PS-II^194,195^ and PS-III^196^ oligosaccharides (Figure 20) were found to be immunogenic in mice when linked to CRM197 carrier protein. Very recently, these semisynthetic glycoconjugates were found to protect from *C. difficile* infections in a murine challenge model more effectively than an antitoxin vaccine candidate.^193^

#### 3.1.4 Shigella

While at present, there are no licensed vaccines available for Shigella, studies in animals and humans have demonstrated that protection by vaccination is feasible. With four major species and 50 different serotypes of Shigella, the development of a broad vaccine may become difficult and expensive.^197^ Serum and mucosal antibody responses to Shigella are predominantly directed against a serotype-specific *Shigella* LPS O-antigen. These robust responses lead to the induction of memory B-cells evidence of their ability to cross-protect against diverse serotypes is inconclusive.^198,199^ After early work focusing on the glycan of *S. dysenteriae* type 1,^200^ the first synthetic oligosaccharide-based conjugate vaccine candidate against the most endemic *Shigella*, *S. flexneri* serotype 2a (SF-2a), was developed.^201^

### 3.2 Improving Existing Vaccines for Problematic Serotypes

#### 3.2.1 Immunogenicity Problems: *S. pneumoniae* Serotype 3

*S. pneumoniae* serotype 3 (SP-3) causes invasive pneumococcal infections in adults and is covered by Prevnar13. The SP-3 glycoconjugates contained in Prevnar13 showed an atypical immunogenicity and boostability pattern and are of relatively low efficacy thus resulting in hyporesponsiveness.^202^ Altered CPS expression, capsule thickness, and an impaired booster response may all contribute to the low efficacy of ST3 conjugates.^203^ Prevnar13 is insufficient at limiting acute otitis media infections caused by SP-3.

Synthetic oligosaccharides based on SP-3 CPS RUs protect immunized mice against lethal systemic challenge with SP-3 pneumococci via nonmucosal routes.^204^ A highly immunogenic SP-3 tetrasaccharide glycoconjugate proved to be immunoprotective against experimental pneumonia caused by transnasal infection with SP-3 strain PN36. Antigenic specificities of anti-SP-3 antibodies were dissected by glycan arrays displaying oligosaccharide fragments including the SP-3 CPS RU. An SP-3 oligosaccharide antigen lead for vaccine development was identified by combining organic synthesis, glycan arrays, glycoconjugation, in vivo vaccination and challenge experiments.^204^

#### 3.2.2 Production Problems: *S. pneumoniae* Serotype 5

*S. pneumoniae* serotype 5 (SP-5) is the fifth most prevalent among more than 90 *S. pneumoniae* serotypes, causing invasive pneumococcal disease among young children globally.^205^ A change in the CPS glycan structure during antigen isolation and purification such that the SP-5 antigens no longer sufficiently resemble the native CPS may compromise vaccine efficacy.^206^ Manufacturing glycoconjugate vaccines such as for ST-5 can be problematic when CPS contain labile groups (see section 2.1.4.).^207^

Marketed glycoconjugate vaccines are manufactured from either native or depolymerized CPS that are typically coupled to a carrier protein via reductive amination following isolation. The keto group present in the rare sugar Sugp (Figure 14) is partially or fully reduced to form a mixture of SP-5 CPS components and degrades during SP-5 glycoconjugate production. The complex CPS thus generated is characterized by variable RUs leading to manufacturing issues and decreased immunogenicity compared to the native SP-5 CPS.^208^

**Figure 21:**
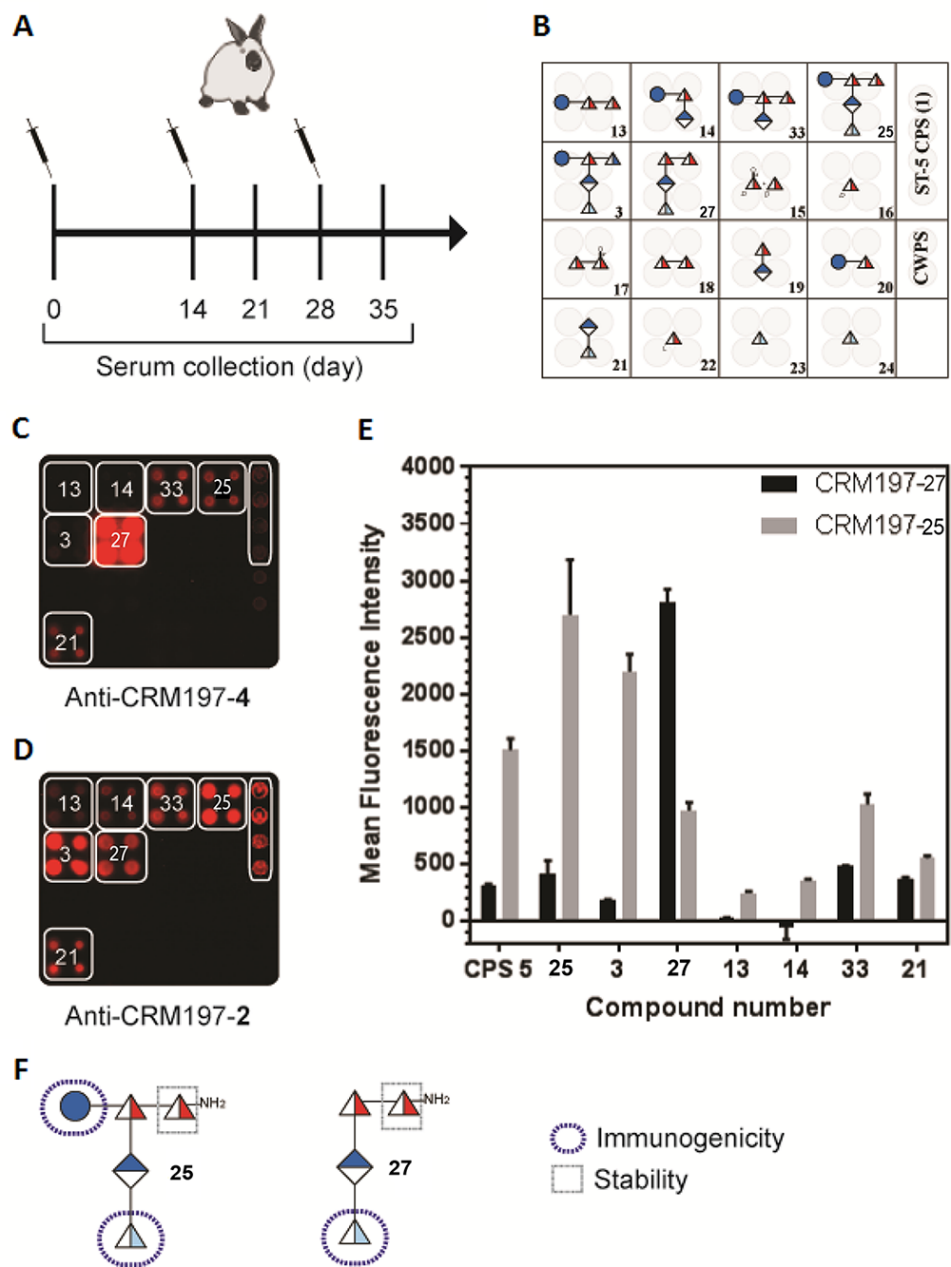
Microarray with rabbit anti-CRM197-**25** and anti-CRM197-**27** conjugates sera. (A) Immunization pattern and sera collection schedule. Rabbits immunized with CRM197-**25** and CRM197-**27** conjugates in a prime boost manner on days 0, 14, and 28. Preimmune and hyperimmune sera collected at different time points from individual rabbits. (B) Microarray slide printing pattern. (C and D) Microarray slides incubated with rabbit sera (day 35) raised against CRM197-**25** and CRM197-**27** conjugates. (E) MFI of cross-reactive spots plotted as mean ± SD in duplicates. (F) Representation of glycans **25** and **27** immunogenicity and stability moieties.

Synthetic SP-5 oligosaccharides (**25**-**27**) resembling the CPS RU provided insights into how changes to the natural SP-5 CPS may influence antigen stability and immunogenicity.^141^ Total synthesis provided access not only to the natural keto containing RU **25**, but also oligosaccharides containing the alcohol products that are the result of the ketone reduction in **25** (Figure 14). Using these synthetic epitopes, vaccine design considerations relating to the effect of branching, length and the role of unique sugars like L-PneuNAc and Sugp on overall immunogenicity and protection were evaluated. The oligosaccharides related to the SP-5 CPS RU containing a reducing end linker were fixed on glycan arrays and conjugated CRM197 (Figure 21).

Glycan array analyses of human reference sera and immunization experiments in rabbits identified the rare aminosugar L-PneuNAc, as well as branching as key to antibody recognition and avidity. Oligosaccharide **25** containing a secondary alcohol in place of the labile ketone in native SP-5 CPS 1 resulted in improved antibody titers and opsonic activity when compared to Prevnar13 that contains natural SP-5 CPS.^141^ The medicinal chemistry approach allowed for the identification of key epitopes and the replacement of nonessential, labile entities that create production problems with closely related, stable functional groups. Synthetic chemistry helps to overcome vaccine manufacturing problems associated with *S. pneumoniae* SP-5 vaccines by increasing stability, immunogenicity, and protection.^141^

### 3.3 Expansion of Marketed Glycoconjugate Vaccines with Synthetic Glycoconjugates

Current polysaccharide-based *S. pneumoniae* glycoconjugate vaccines such as Prevnar13 or Synflorix do not cover all medically important serotypes and some serotypes that are included in existing vaccines are either poorly immunogenic or face production problems. The addition of a semisynthetic glycoconjugate may expand current formulations or help to replace serotypes that are not efficiently targeted due to difficulties during polysaccharide isolation and conjugation.

#### 3.3.1 Addition of Semisynthetic SP-8 Glycoconjugate to Prevnar 13

**Figure 22:**
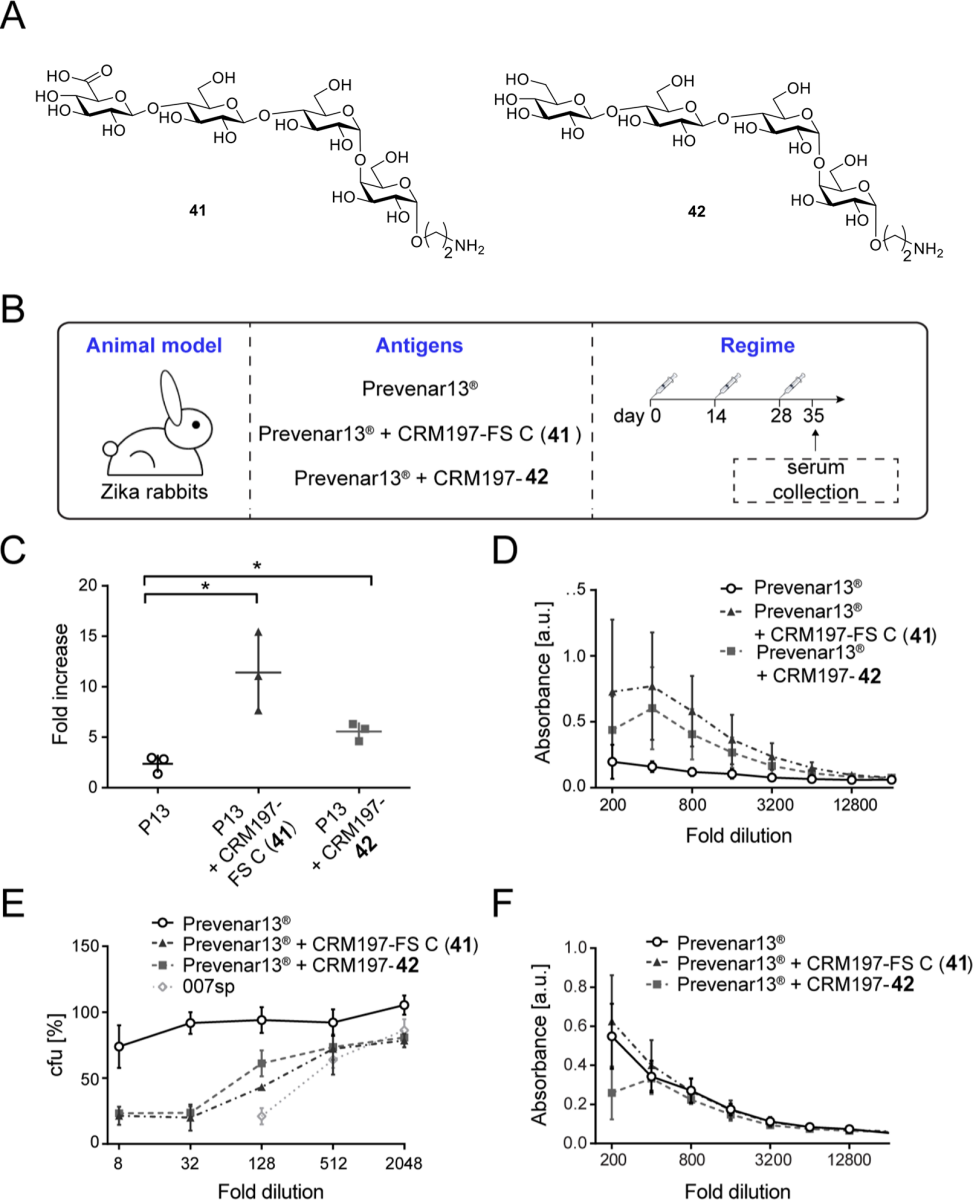
Co-formulation of semisynthetic SP-8 glycoconjugates and Prevnar13. (a) SP-8 glycoconjugates CRM197-**41** and CRM197-**42** were added to Prevnar13. (b) Rabbits were immunized three times with Prevnar13 with or without coadsorbed CRM197 glycoconjugates. Serum was collected at day 35. (c) Evaluation of ST8 CPS binding by rabbit sera at day 35. (d) Comparison of SP-8 CPS binding of rabbit sera from different groups. (e) Comparison of opsonophagocytic killing of SP-8 pneumococci by pooled rabbit sera. (f) Comparison of opsonophagocytic killing by pooled rabbit sera and human reference serum 007sp at different serum dilutions.

*S. pneumoniae* serotype 8 is not contained in Prevnar13. To assess whether semisynthetic oligosaccharide-based SP-8 glycoconjugates are compatible with marketed polysaccharide-based vaccines, SP-8 glycoconjugate was coformulated with Prevnar13 to create a 14-valent vaccine.^133^ Adsorption of tetrasaccharide 22-CRM197 glycoconjugates to the aluminum phosphate-based Prevnar13 emulsion was confirmed by flow cytometry, using mAb 1H8 to detect these glycans (Figure 22). SP-8 glycoconjugates were adsorbed to Prevnar13 in a dose-dependent manner (Figure 22). SP8 glycoconjugate adsorption did not abrogate adsorption of other serotypes in Prevnar 13.^133^

Immunization of rabbits with two 14-valent coformulations of Prevnar13 and CRM197-**41** or CRM197-**42** with a glycan dose equal to that of most individual serotypes in the commercial vaccine induced a pronounced immune response against SP8 CPS (Figure 22c,d). Sera of rabbits immunized with the 14-valent vaccines mediated opsonophagocytic killing of SP-8 pneumococci, in contrast to sera from Prevnar13-immunized rabbits (Figure 22e,f). Co-formulation with SP-8 glycoconjugates did not impair the immunogenicity of other Prevnar13-specific glycoconjugates, as the immune response of rabbits toward six Prevnar13-specific CPSs did not decrease. The, semisynthetic SP-8 glycoconjugates were found to induce a robust, antibacterial immune response when coformulated with a multivalent, polysaccharide-based glycoconjugate vaccine.^133^

#### 3.3.2 Addition of Multiple Semisynthetic Glycoconjugates to Prevnar 13

**Figure 23:**
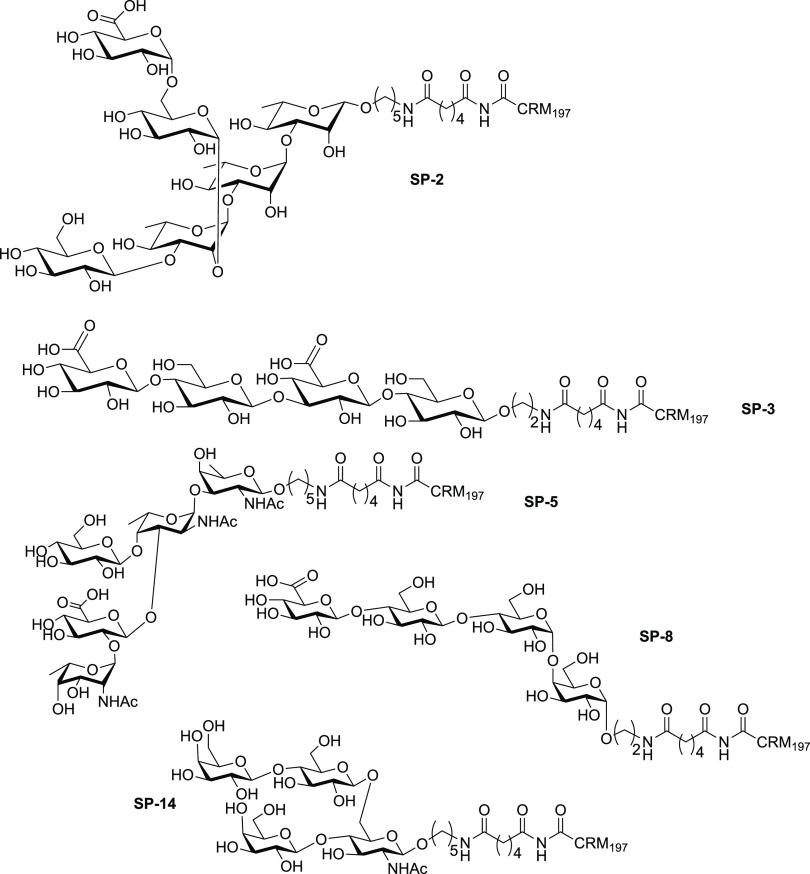
CRM197 conjugates of synthetic oligosaccharide antigens resembling the CPS of *S. pneumoniae* serotypes 2 (SP-2), 3 (SP-3), 5 (SP-5), 8 (SP-8), and 14 (SP-14).

**Figure 24:**
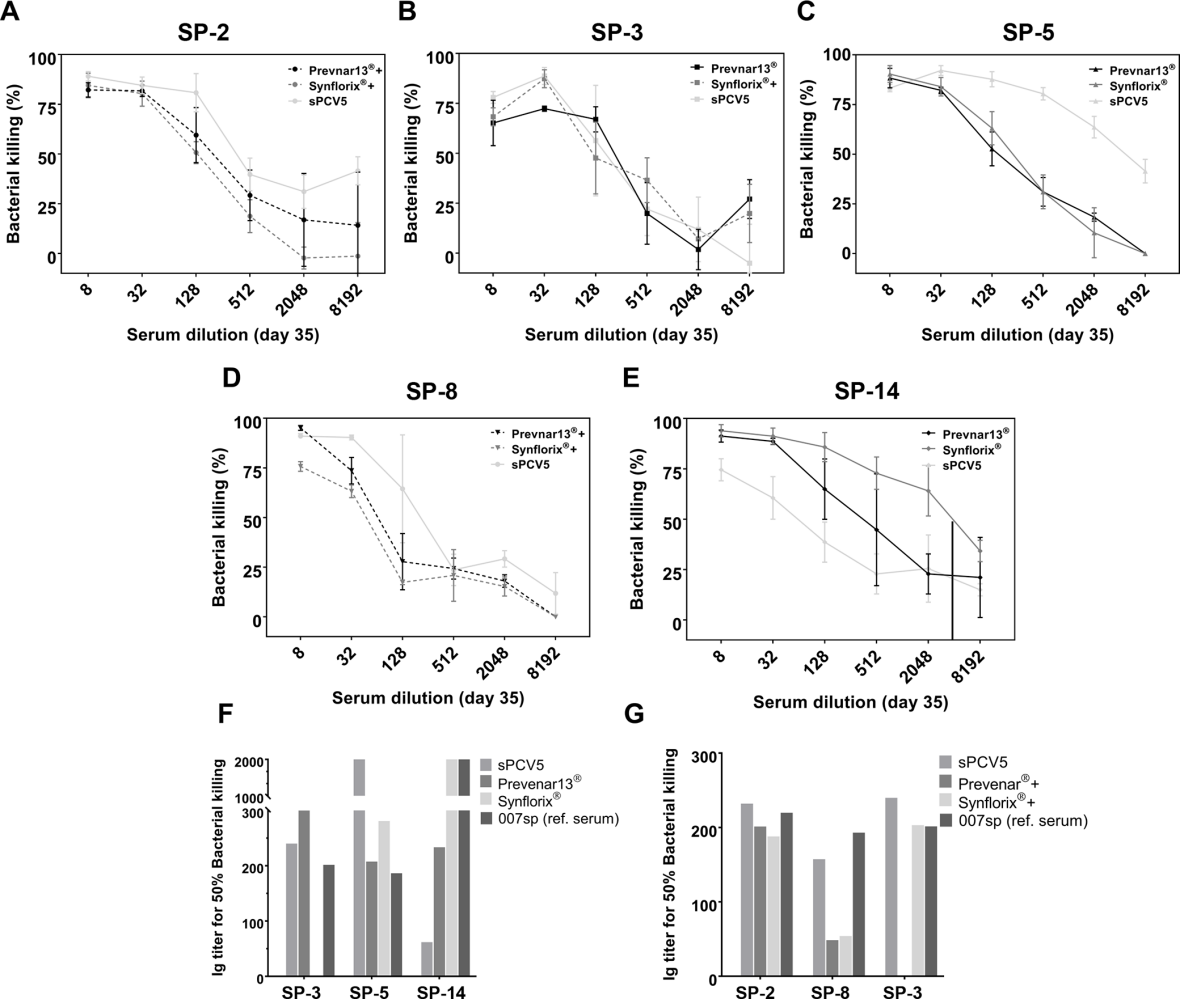
In vitro opsonophagocytic activity of a vaccine formulation containing five synthetic oligosaccharide antigens (sPCV5). (a–e) Comparison of pooled sera from rabbit immunized with sPCV5, Prevnar13+SP-2+SP-8, Synflorix+SP-2+SP-3+SP-8 as well as positive controls Prevnar13 and Synflorix for opsonophagocytic killing of *S. pneumoniae* serotypes (A) SP-2, (B) SP-3, (C) SP-5, (D) SP-8, and (E) SP-14. (f and g) Comparison of antibody titer of pooled serum responsible for 50% killing of bacteria in opsonophagocytic killing assay. IgG titers are expressed as the reciprocal serum dilution mediating 50% bacterial killing, estimated through nonlinear interpolation of the dilution-killing OPKA data. Human reference serum 007sp was used as a control. Reprinted with permission from ref (181). Copyright National Academy of Sciences 2018.

In order to expand the serotype coverage of marketed Prevnar13 and Synflorix glycocconjugate vaccines a combination of isolated CPS and synthetic oligosaccharides to fix problems and expand to serotypes that are currently not covered. Five synthetic oligosaccharide antigens resembling SP-2, SP-3, SP-5, SP-8, and SP-14 were conjugated to CRM197 carrier protein to test such an approach (Figure 23). Each of those glycoconjugates were separately added to Prevnar13 to create 14-valent vaccines and all five glycoconjugates were also added to Prevnar13 to fix and expand the existing blockbuster vaccine. Immunological evaluation of the coformulated vaccine candidates in rabbits showed encouraging results (Figure 24).^181^

#### 3.3.3 Fully Synthetic Pentavalent Conjugate *S. pneumoniae* Vaccine Candidate

A five-valent vaccine candidate was created by combining five synthetic oligosaccharide antigens representing SP-2, SP-3, SP-5, SP-8, and SP-14 were conjugated to CRM197 and formulating them with the adjuvant alum. The candidate was tested in rabbits and induced a strong immune response against all of the antigens (Figure 24).^181^

## 4 Conclusions and Perspectives

Polysaccharide glycoconjugate vaccines are saving millions of lives each year by protecting children and adults from bacterial infections caused by *H. influenza type b*, *N. meningitidis*, and *S. pneumoniae*. From the first insights about the protective power of isolated polysaccharides and glycoconjugates with protein carriers in the early 20th century, polysaccharide vaccines emerged as an effective alternative to antibiotic treatment. Eventually, these polysaccharide vaccines were replaced by carbohydrate conjugate vaccines that induced a protective T-cell mediated response also in infants, the most important risk group for many infectious diseases. Todaýs marketed glycoconjugate vaccines are almost entirely based on isolated glycans obtained from bacteria. These vaccines are an immense medical and commercial success but the development of additional such glycoconjugate vaccines has proven time and resource consuming.

With the development of automated glycan assembly, access to defined synthetic oligosaccharides has been greatly accelerated such that the desired compounds are obtained in hours or days rather than months of years previously. Quick access to glycan epitopes enables a medicinal chemistry approach to glycoconjugate vaccine development. The minimal protective glycotope of a polysaccharide found on the surface of a pathogenic bacterium or parasite can be identified by preparation of a host of glycans resembling the natural glycan. Using the synthetic glycans, tools such as glycan arrays are prepared in order to interrogate the human immune response to pathogens to glean insights into potential motifs that result in a protective immune response. Immunology, in the interplay with medicinal chemistry is revealing lead structures for further development. We are currently beginning to learn the rules concerning what constitutes an immunogenic and protective antigen. Many factors including antigen length, terminal building block, branching, the presence of unusual sugars and modifications of the glycan are being investigated. While the process of molecular glycoimmunology is still in the early stages, it provides the data that will eventually allow for the design of vaccine antigens rather than the current repetitive trial and error process.

As basic research into glycoimmunology is ongoing, a number of synthetic glycotopes that serve as vaccine candidates have been identified. Semisynthetic glycoconjugates, where a synthetic glycan has been conjugated to a carrier protein such as CRM197 that is already a part of marketed vaccines, have shown great promise in challenge experiments in several animal species. Currently, such semisynthetic glycococonjugate vaccine candidates are in preclinical and clinical development for bacterial diseases including *S. pneumoniae*, *K. pneumoniae*, *C. difficile*, and Shigella. Fully synthetic glycoconjugates where the carrier protein has been replaced by a synthetic glycolpid, peptide, or other carrier are being tested by different laboratories and show much promise.

In the future, the semisynthetic and fully synthetic vaccine candidates are expected to either develop into stand-alone vaccines or to become a part of multivalent vaccines where isolated and synthetic glycans will be combined. The medicinal chemistry approach to glycoconjugate vaccine design and development is complementary to existing approaches based on isolation of glycans but holds great potential to improve existing vaccines and develop completely new modes of protecting humans from our bacterial foes.
